# Cattle and human organoids reveal 2.3.4.4b H5N1 cross-species transmission potential and neuraminidase-specific neutralizing antibodies in humans

**DOI:** 10.1038/s41467-026-74345-w

**Published:** 2026-07-08

**Authors:** Cun Li, Yifei Yu, Zhixin Wan, Jian-Piao Cai, Jingjing Huang, Xiaoxin Zhu, Jiali Wu, Wei Xue, Ying Zhou, Man Chun Chiu, Qiaoshuai Lan, Shuxin Zhang, Zijun Zhao, Yidong Yang, Yuhong Liu, Pui Wang, Shaofeng Deng, Ming Yue, Longchao Zhu, Keshan Zhang, Mengmeng Zhao, Jieshi Yu, Wenkang Wei, Xiang Gao, Lin Huang, Hin Chu, Gang Wu, Honglin Chen, Kwok Yung Yuen, Richard Webby, Jie Zhou

**Affiliations:** 1 Department of Microbiology, School of Clinical Medicine, Li Ka Shing Faculty of Medicine, The University of Hong Kong, Hong Kong Special Administrative Region, China; 2 Centre for Virology, Vaccinology and Therapeutics, Hong Kong Science and Technology Park, Hong Kong Special Administrative Region, China; 3 School of Life and Health Technology, Dongguan University of Technology, Dongguan, China; 4 Guangdong Provincial Key Laboratory of Animal Molecular Design and Precise Breeding, School of Animal Science and Technology, Foshan University, Foshan, China; 5 State Key Laboratory of Swine and Poultry Breeding Industry, Agro-Biological Gene Research Center, Guangdong Academy of Agricultural Sciences, Guangzhou, China; 6 Laboratory of Advanced Biotechnology, Beijing Institute of Biotechnology, Beijing, China; 7 BiomOrgan Ltd, Hong Kong, China; 8 State Key Laboratory of Emerging Infectious Diseases, Li Ka Shing Faculty of Medicine, The University of Hong Kong, Hong Kong Special Administrative Region, China; 9 Pandemic Research Alliance Unit at The University of Hong Kong, Hong Kong Special Administrative Region, China; 10 Department of Host Microbe Interactions, St. Jude Children’s Research Hospital, Memphis, TN USA; 11 Carol Yu Centre for Infection, Li Ka Shing Faculty of Medicine, The University of Hong Kong, Hong Kong Special Administrative Region, China; 12 Department of Infectious Disease and Microbiology, The University of Hong Kong-Shenzhen Hospital, Shenzhen, China; 13 Department of Microbiology, Queen Mary Hospital, Pokfulam, Hong Kong Special Administrative Region, China

**Keywords:** Influenza virus, Adult stem cells

## Abstract

The unexpected circulation of clade 2.3.4.4b H5N1 influenza viruses in dairy cattle and the transmission to diverse mammalian species poses a pandemic risk. We sought to explore cattle and human respiratory susceptibility to the 2.3.4.4b H5N1 virus. We establish long-term expandable cattle airway and mammary organoids. The 2.3.4.4b H5N1 virus exhibits high replicative fitness in cattle mammary organoids, recapitulating its remarkable mammary tropism. The virus also replicates robustly in cattle airway organoids, suggesting an underrecognized respiratory component in ongoing outbreaks. Interestingly, human airway and nasal organoids are highly susceptible to the 2.3.4.4b H5N1 virus. Yet, a novel organoid-based neutralization assay reveals that N1 antibodies in human sera had cross-neutralizing activity against the 2.3.4.4b H5N1 and ancestral H5N1-VN1194 viruses. The cross-neutralization, exclusively manifested in the organoid-based assay, is enhanced after seasonal influenza vaccination and diminished after depleting N1-specific antibodies. Therefore, cross-neutralizing N1 antibodies are likely limiting zoonotic infection by H5N1 viruses in humans.

## Abstract (web summary)

Active 2.3.4.4b H5N1 infection in cattle airway organoids suggests respiratory transmission in dairy farms. The virus also replicates in human respiratory organoids, yet pre-existing N1-specific antibodies may protect humans against H5N1 infection.

## Introduction

Highly pathogenic avian influenza A(H5N1) viruses have emerged and circulated in wild and domestic birds since the late 1990s; the first outbreak of fatal H5N1 human infection was reported in Hong Kong in 1997^1^. Since 2021, H5N1 viruses of clade 2.3.4.4b have spread globally, infecting diverse avian and mammalian species at an alarming pace^2–5^. In March 2024, an outbreak of the 2.3.4.4b H5N1 virus was reported in dairy cattle in Texas, with subsequent spread to hundreds of farms and cross-species infections in mammals, including humans^6^. At least 70 confirmed human cases of 2.3.4.4b H5N1 infection with two fatalities in North America have been documented from April 2024 to May 2025, underscoring the public health risk posed by this evolving clade of viruses^7,8^. Fortunately, the infected individuals predominantly presented with conjunctivitis and mild respiratory symptoms^8^.

Mammary infection with clade 2.3.4.4b H5N1 viruses has been extensively documented in naturally infected dairy cattle. The field-infected animals exhibited a marked decline in milk yield with abnormal milk, reduced feed intake, and, in some cases, respiratory distress^6,9^. Mammary infections with 2.3.4.4b H5N1 viruses were reported after direct experimental intramammary inoculation in lactating cows, which had decreased milk production with evidence of replicating viruses in the mammary glands^10^. All inoculated cattle showed evidence of respiratory infection after intranasal inoculation with two avian H5N1 viruses closely related to the 2.3.4.4b virus detected in cattle^11^. Moreover, increased nasal secretions and virus infection of airway epithelial cells were observed in heifers (young female cows) after aerosol inoculation^10^. Currently, it is believed that the viruses are mainly transmitted on dairy farms via mammary infection due to the shared use of milking equipment. However, the evidence of cattle respiratory infection raises the possibility of respiratory transmission in dairy farms, which is indeed a common transmission route of influenza viruses in mammals. This hypothesis is supported by ongoing outbreaks and persistent transmission, despite enhanced surveillance efforts, including cattle farm monitoring and milk testing^12^. These findings, collectively, imply that cattle 2.3.4.4b H5N1 respiratory infection and potential respiratory transmission may be underrecognized and warrant further investigation.

Fortunately, human 2.3.4.4b virus infection in the U.S. is unexpectedly mild, which also notably differs from the highly virulent and systematic infections observed in mice and ferrets after intranasal inoculation with the bovine H5N1 virus^13^. A bovine H5N1 isolate from an infected farm worker caused lethal infection in mice and ferrets^14^. One possible explanation for the contradictory disease presentation is the prevalence of cross-reactive antibodies targeting the neuraminidase (NA) of 2.3.4.4b viruses in healthy humans^2^. However, while the potency of HA-targeting antibodies to inhibit influenza virus entry is routinely measured in standard cell line-based neutralization assays, the neutralizing potential of NA antibodies cannot be manifested in these conventional assays^15,16^. There has been a long-awaited need to evaluate the neutralizing effect of NA antibodies, and specifically, herein, the potential protection of pre-existing cross-reactive NA antibodies against the emerging 2.3.4.4b H5N1 viruses.

We have developed a human respiratory organoid culture system, which has enabled us to derive organoids from nasal cells and primary lung tissues with high efficiency and generate nasal, airway, and alveolar organoids^17–19^. The nasal organoids are established from nasal epithelial cells collected from volunteer donors noninvasively; they sustain long-term expansion for over half a year, similar to the organoids derived from primary lung tissues. Upon induction of maturation, we generated differentiated nasal organoids and airway organoids from the long-term expandable organoids. The mature nasal and airway organoids contain four major types of airway epithelial cells; while maintaining compartment-specific transcriptomic signatures^18^. These respiratory organoids have become robust and biologically relevant in vitro models for studying respiratory viruses^20^. Recently, we reported the development of nasal organoid-based SARS-CoV-2 neutralization assays, which revealed a distinct neutralization profile of SARS-CoV-2 monoclonal antibodies compared to the conventional cell-line-based neutralization assays and recapitulated their real-world neutralizing efficacy^21^. We also developed the first bat intestinal organoids, thereby providing the first wet-lab evidence for the bat origin of SARS-CoV-2^22^.

In this study, we established cattle airway and mammary organoids, aiming to gain an in-depth understanding of 2.3.4.4b H5N1 virus tropism in the cattle respiratory tract and mammary tissues. Moreover, we assessed the susceptibility of human airway and nasal organoids to the cattle 2.3.4.4b H5N1 virus and examined the potential of pre-existing cross-reactive NA antibodies in protecting humans from 2.3.4.4b virus infection.

## Results

### Establishment and characterization of cattle airway and mammary organoids

**Fig. 1:**
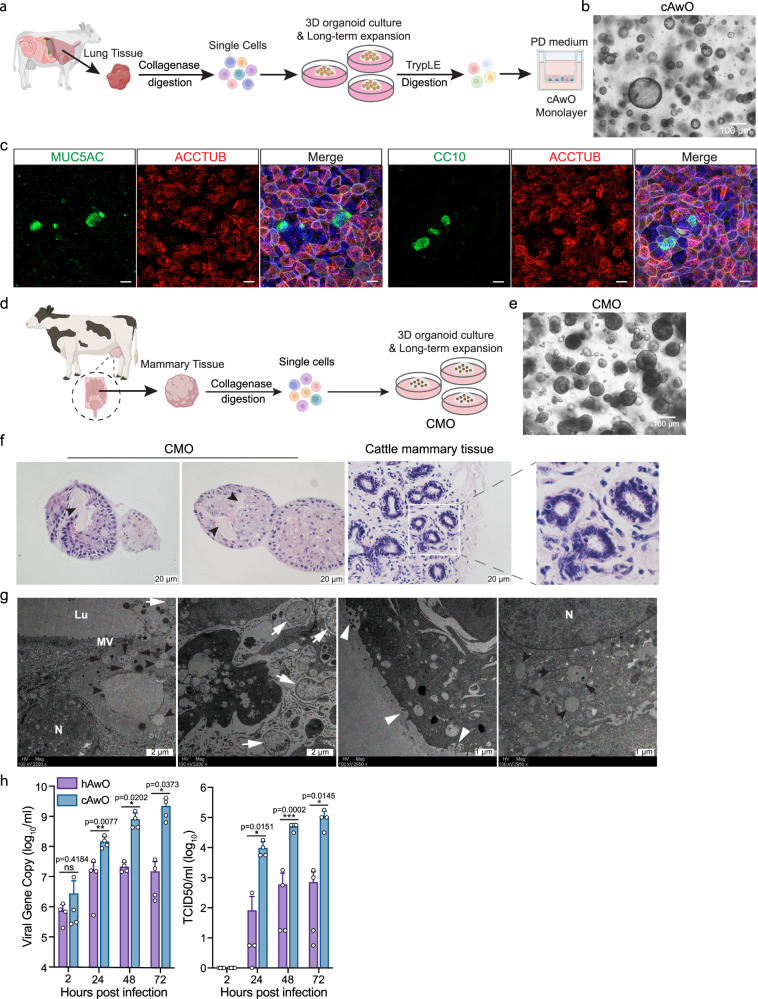
Establishment and characterization of dairy cattle airway and mammary organoids. **a** The diagram illustrates the derivation of cattle airway organoids (cAwO) from cattle lung tissues, expansion culture and differentiation culture to generate mature airway organoid monolayers in transwell plates, Created in BioRender. CVVT, C. (2026) https://BioRender.com/s6vm9vp. **b** Cattle lung tissue-derived organoids undergo expansion in 3D culture. The brightfield image shows growing organoids on day 7. **c** Cattle airway organoid monolayers were subjected to immunofluorescence staining to label MUC5AC+ goblet cells (green), CC10+ club cells (green), and ACCTUB+ ciliated cells (red). Nuclei and actin filaments were counterstained with DAPI (blue) and Phalloidin-647 (white), respectively. Scale bar, 10 μm. **d** The diagram illustrates the generation of cattle mammary organoids (CMO) from cattle udder tissues, Created in BioRender. CVVT, C. (2026) https://BioRender.com/d6rj7m3. **e** The brightfield image shows the expanding organoids on day 20. **f** Photomicrographs of hematoxylin and eosin staining of cattle mammary organoids (left) and cattle mammary tissue (right). The black arrowhead indicates the accumulation of proteinaceous secretions within the CMO. **g** Transmission electron microscopy demonstrates characteristic organelles in cattle mammary organoids. Golgi vesicles (black arrow) and lipid droplets (black arrowhead) aggregate on the apical side of the luminal cells. The white arrowhead indicates the site of the Golgi vesicle exocytosis from the cell membrane. The secreted milk fat globule (MFG) in the lumen is marked with a white arrow. Abbreviations: Lu Lumen, N nucleus, MV Microvilli. The experiments in Fig. 1b, c, e–g were independently repeated three times with similar results. **h** Human airway organoids (hAwO) and cattle airway organoids were inoculated with influenza D viruses (IDV) at 0.01 multiplicity of infection (MOI) (*n* = 4). Culture media were harvested from infected organoids at the indicated hours post-inoculation and applied to the detection of viral replication by RT-PCR and TCID50. Statistical significance was determined using a two-tailed Student’s *t*-test. Data represent mean and SD of the indicated number (*n*) of biological replicates. *P  <  0.05, **P  <  0.01, ***P  <  0.001. Source data are provided as a Source data file for Fig. 1h.

We sought to establish cattle airway and mammary organoids, which would enable us to conduct an in-depth investigation of 2.3.4.4b H5N1 virus tropism in the respiratory and mammary tissues. Organoids were derived from cattle lung tissues, following a protocol to generate human respiratory organoids^19,23^ (Fig. 1a). Briefly, single cells dissociated from cattle lung tissues were embedded in Matrigel and maintained in a respiratory organoid expansion medium^19,20^. The derived 3D organoids were directed toward an immature state during serial passage over a period of 6 months (Fig. 1b). We seeded the organoids onto transwell inserts and incubated them with a proximal differentiation (PD) medium to induce maturation^24^. The monolayers of differentiated cattle airway organoid (thereafter cattle airway organoids, cAwO) contained airway epithelial cell types, including ACCTUB+ ciliated cells, MUC5AC+ Goblet cells, and CC10+ Club cells (Fig. 1c). Ciliated cells, the major cell population in the airway epithelium, were notably enriched in the cattle airway organoids (Fig. 1c), similar to human airway organoids^19^.

The mammary gland is the characteristic tissue of mammals. The bi-layered mammary alveoli consist of two major cell types, cuboidal luminal cells lining the alveoli and producing milk, and the underlying basal cells. We generated 3D cattle mammary organoids (CMO) from the mammary tissue of a Holstein–Friesian cow using the method for deriving human breast organoids as described previously^25,26^ (Fig. 1d). At the time of paper writing, the derived organoids had undergone 15 passages for 6 months. The expanding mammary organoids were solid spheres or spheres with a central lumen (Fig. 1e). Given that luminal cells were the primary target of cattle H5N1 viruses^6^, we generated mammary organoids with enriched luminal cells by removing EGF from the culture medium, an approach previously described in human breast organoids^26^. The resultant organoids were applied to subsequent characterization and infection experiments. These mammary organoids maintained in Matrigel exhibited an apical-in polarity^27,28^ and displayed a gland-like architecture with pinkish proteinaceous secretion from the luminal cells within the lumen, morphologically similar to the mammary glands in cattle mammary tissue (Fig. 1f). Transmission electron microscopy revealed microvilli (MV), Golgi vesicles (GV), lipid droplets (LDs), and milk fat globule (MFG) in the cytosol, which are the characteristic organelles in native mammary luminal cells (Fig. 1g). The exocytosis of Golgi vesicles, previously shown in native mammary epithelial cells of cattle and other animals^29^, was readily discernible on the apical side of luminal cells within cattle mammary organoids (Fig. 1g). Overall, the derived cattle airway and mammary organoids faithfully simulate the cellular composition and morphology of the cattle airway epithelium and mammary gland, respectively.

Influenza A viruses (IAVs) are the etiological agents of human influenza infections^30^, whereas influenza D viruses (IDVs) primarily infect cattle that are normally not susceptible to IAVs. IDV-infected cattle developed mild to moderate respiratory clinical signs, including nasal discharge, cough, and dyspnea^30^. To further verify the biological relevance of cattle airway organoids, we inoculated an IDV isolate in cattle airway organoids and examined viral growth; human airway organoids (hAwO) were inoculated with the same IDV in parallel for comparison. A significant increase in viral gene copy and viral titer was detected in the culture media of cattle airway organoids (Fig. 1h). Viral titer increased by 5 log units 72 h post-infection, indicating a robust viral replication. In contrast, the same virus grew modestly in human airway organoids. Viral titer was 2–3 log units higher in cattle airway organoids than in human counterparts (Fig. 1h). On the other hand, human airway organoids sustained robust replication of a seasonal (H1N1)pdm09 human influenza A virus, with a dramatically increased viral titer of 7–8 log units (Supplementary Fig. 1) as we described previously^19,24^. Overall, we developed novel, biologically relevant cattle airway and mammary organoids. More importantly, the cattle airway organoids adequately recapitulated the host-specificity of IDV infection in cattle.

### Robust 2.3.4.4b H5N1 virus replication in cattle airway and mammary organoids

**Fig. 2:**
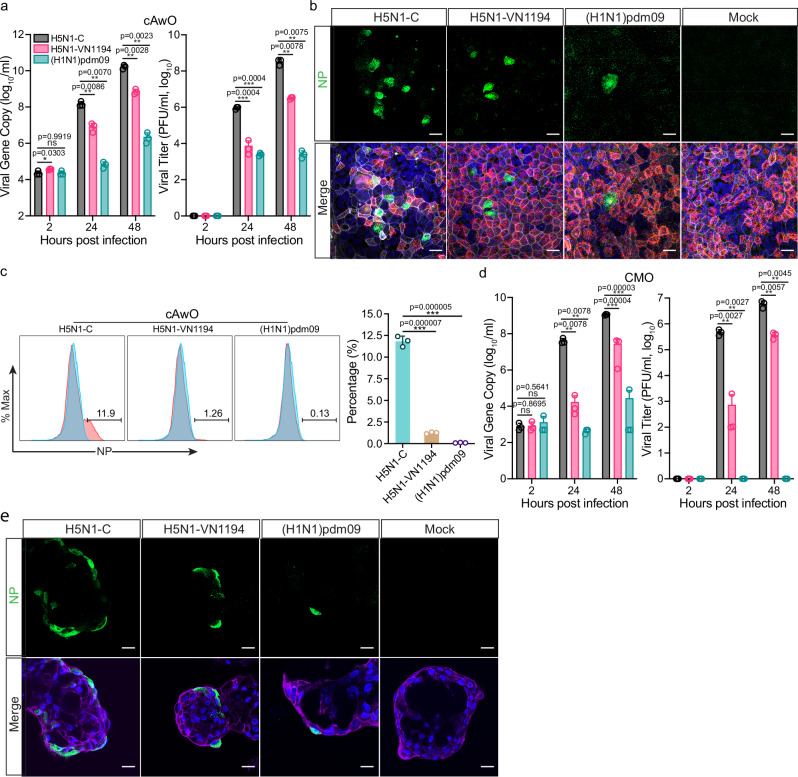
The robust replication of 2.3.4.4b H5N1 in cattle airway and mammary organoids. **a**–**c** Cattle airway organoids (cAwO) were inoculated with 2.3.4.4b H5N1 (H5N1-C), an avian H5N1 (H5N1-VN1194), and H1N1 pandemic ((H1N1)pdm09) viruses at 0.01 MOI. **a** Culture media were harvested from infected organoids at the indicated hours post-inoculation and applied to the detection of viral replication by RT-PCR and plaque assay (*n* = 3). Data represent mean and SD of the indicated number (*n*) of biological replicates. Statistical significance was determined using a two-tailed Student’s *t*-test. *P < 0.05, **P < 0.01, ***P < 0.001. **b** At 24 h post-inoculation (hpi), the infected organoids were fixed and applied to immunostaining for influenza viral NP+ (green) and ACCTUB+ ciliated cells (red). Nuclei and actin filaments were counterstained with DAPI (blue) and Phalloidin-647 (white), respectively. Scale bar, 10 µm. **c** H5N1-C-, H5N1-VN1194-, and (H1N1)pdm09-infected cattle airway organoids were fixed after dissociation, then applied to immunostaining and flow cytometry to detect the percentage of virus-infected cells (*n* = 3). Data represent mean and SD of the indicated number (*n*) of biological replicates. Statistical significance was determined using a two-tailed Student’s *t*-test. ***P < 0.001. **d, e** Cattle mammary organoids (CMO) were inoculated with H5N1-C, H5N1-VN1194, and (H1N1)pdm09 viruses at 0.1 MOI. **d** Culture media were harvested from infected organoids at the indicated time points for viral load detection and viral titration by plaque assay (*n* = 3). Data represent mean and SD of the indicated number (*n*) of biological replicates. Statistical significance was determined using a two-tailed Student’s *t*-test. *P < 0.05, **P < 0.01, ***P < 0.001. **e** The infected organoids were fixed and applied to immunostaining for influenza viral NP+ (green) and ACCTUB+ ciliated cells (red) at 24 hpi. Nuclei and actin filaments were counterstained with DAPI (blue) and Phalloidin-647 (white), respectively. Scale bar, 10 µm. The experiments in Fig. 2b, e were independently repeated three times with similar results. Source data are provided as a Source data file for Fig. 2a, c, and d.

We next inspected the tissue tropism of a cattle-infective H5N1 virus in cattle airway and mammary organoids. We constructed the 2.3.4.4b H5N1 virus (H5N1-C) by reverse genetics based on the genetic sequence of A/dairy cattle/Texas/24-008749-003/2024 (H5N1)^31^ and examined its replication kinetics in cattle airway organoids. A reference Clade 1 H5N1 virus (A/Vietnam/1194/2004 H5N1, H5N1-VN1194)^32^, and the human (H1N1)pdm09 virus (A/Hong Kong/415742/2009) were used as comparators. After infections at an MOI of 0.01, H5N1-C replicated to a significantly higher level than H5N1-VN1194 in cattle airway organoids (Fig. 2a). The viral titer of H5N1-C reached approximately 8 log units/ml within 48 h post-infection, over 2 log units higher than that of H5N1-VN1194, and over 5 log units higher than that of (H1N1)pdm09 (Fig. 2a, right). To further characterize the infectivity of these viruses, we inoculated the cattle airway organoids with H5N1-C, H5N1-VN1194, and (H1N1)pdm09 at an MOI of 0.01, followed by immunostaining and confocal imaging 24 h after infections. There were more abundant NP+ cells in H5N1-C infected airway organoids than those inoculated with H5N1-VN1194 (Fig. 2b). In the mock-infected airway organoid monolayers, abundant ciliated cells with dense ACCTUB+ cilia were evenly distributed in organoid monolayers (Fig. 2b); whereas the cilia were remarkably depleted in virus-infected organoids, consistent with the cellular tropism of respiratory viruses in human airway organoids^17,18,20,24^. Moreover, ciliary depletion was more prominent in organoids infected with H5N1-C than those infected with H5N1-VN1194 (Fig. 2b). The human (H1N1)pdm09 virus showed minimal infection in the cattle airway organoids (Fig. 2b). Flow cytometry analysis verified the significantly higher infection rate of H5N1-C compared to H5N1-VN1194 in the cattle airway organoids, with few positive cells present in (H1N1)pdm09-infected organoids (Fig. 2c, Supplementary Fig. 2).

We next analyzed the replication kinetics of the three virus strains in cattle mammary organoids. After mechanical disruption, CMOs were inoculated at an MOI of 0.01, followed by suspension culture. H5N1-C reached a viral titer of 7 log units/ml at 48 hpi, around 1 log unit higher than H5N1-VN1194 (Fig. 2d). (H1N1)pdm09 showed minimal infectivity, with detectable genomic RNA but no infectious particles, suggesting a non-productive infection (Fig. 2d). Imaging analysis of cattle mammary organoids verified the higher infectivity of H5N1-C than H5N1-VN1194 and (H1N1)pdm09 (Fig. 2e). The NP+ cells were located at the periphery of the infected organoids (Fig. 2e), since the suspension culture resulted in an apical-out polarity in epithelial organoids as reported previously^27,28^. Collectively, the high replicative fitness of H5N1-C in cattle mammary organoids recapitulated the unprecedented mammary tropism of H5N1 2.3.4.4b in field-infected and experimentally infected cattle. The notably increased replicative fitness of 2.3.4.4b H5N1 over the ancestral H5N1-VN1194 virus in cattle mammary and airway organoids indicates that the ability to infect dairy cattle might be a newly acquired attribute of recent H5N1 viruses. More importantly, robust H5N1-C replication in cattle airway organoids suggests that an under-recognized respiratory transmission may drive virus spread.

### Susceptibility and cellular response of human airway and nasal organoids to 2.3.4.4b H5N1 virus

**Fig. 3:**
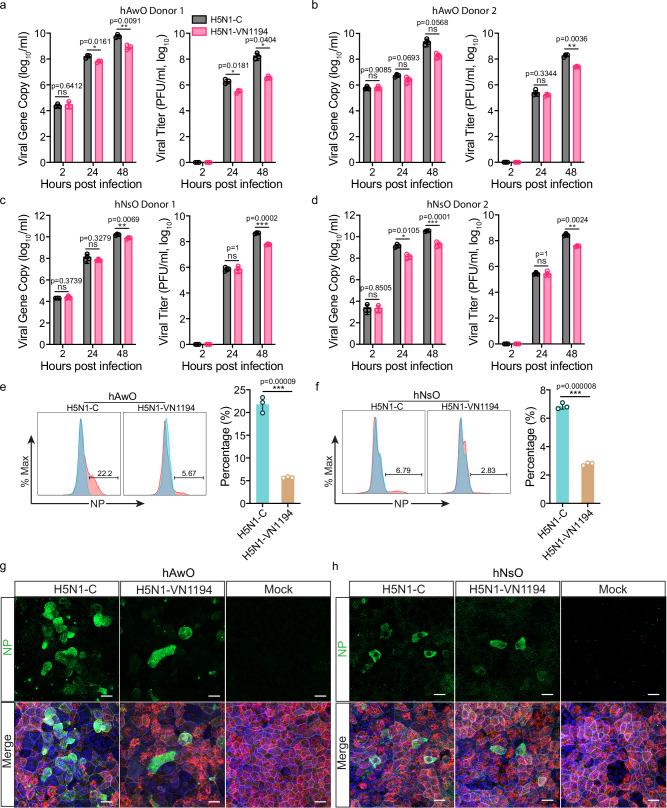
The high infectivity and replication fitness of 2.3.4.4b H5N1 in human airway and nasal organoids. Human airway (**a**, **b**) and nasal organoids (**c**, **d**) were inoculated with H5N1-C and H5N1-VN1194 at 0.01 MOI (**a**, **d**, *n* = 3; **b**, **c**, *n* = 4). Culture media were harvested from infected organoids at the indicated time points for viral load detection and viral titration by plaque assay. Data represent mean and SD of the indicated number (*n*) of biological replicates. Statistical significance was determined using a two-tailed Student’s *t*-test. *P < 0.05, **P < 0.01, ***P < 0.001. Human airway (**e**) and nasal (**f**) organoids were infected with H5N1-C and H5N1-VN1194 at 0.01 MOI, respectively. After dissociation, these organoids were fixed and subsequently applied to immunostaining and flow cytometry to determine the percentage of virus-infected cells (*n* = 3). Data represent mean and SD of the indicated number (*n*) of biological replicates. Statistical significance was determined using a two-tailed Student’s *t*-test. ***P  <  0.001. Human airway (**g**) and nasal (**h**) organoids were inoculated with H5N1-C and H5N1-VN1194 at 0.01 MOI. The infected organoids were fixed at 24 hpi and applied to immunostaining of influenza viral NP (green) and ACCTUB+ ciliated cells (red). Nuclei and actin filaments were counterstained with DAPI (blue) and Phalloidin-647 (white), respectively. Scale bar, 10 µm. The experiments in g and h were independently repeated three times with similar results. Source data are provided as a Source data file for (**a**–**f**).

To model human susceptibility to H5N1 virus infection, we examined the infectivity and replicative fitness of H5N1-C and H5N1-VN1194 in human airway organoids. H5N1-C actively replicated in airway organoids, reaching a viral titer of over 8 log units/ml in the culture medium 48 h post-infection at an MOI of 0.01 (Fig. 3a). H5N1-VN1194 replicated less robustly, which was verified in airway organoids derived from a second donor (Fig. 3b). While H5N1-C consistently outgrew H5N1-VN1194 in airway organoids from two donors (Fig. 3a, b), the growth advantage varied between the two lines. A higher replication capacity of H5N1-C than H5N1-VN1194 was also observed in human nasal organoids derived from two additional donors, an organoid model of the upper human airways (Fig. 3c, d). A significantly higher percentage of NP+ cells was seen in H5N1-C-infected airway and nasal organoids than in those infected with H5N1-VN1194, as revealed by flow cytometry analysis and confocal imaging (Fig. 3e–h and Supplementary Fig. 2). Overall, H5N1-C exhibited a significantly higher replication fitness than H5N1-VN1194 in human nasal and airway organoids.

**Fig. 4:**
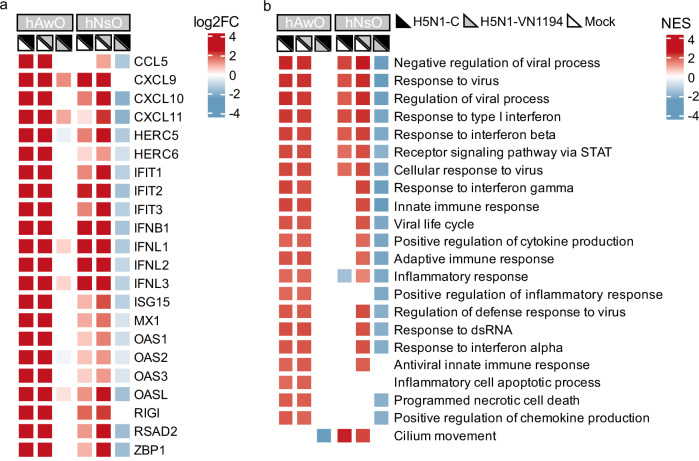
Cellular response to 2.3.4.4b H5N1 and H5N1-VN1194 in human airway and nasal organoids. H5N1-C-, H5N1-VN1194-, and mock-infected airway and nasal organoids were applied to RNA sequencing analysis. **a** The heatmap depicts log2FC (color) of cytokines and ISGs in the virus- and mock-infected airway and nasal organoids. **b** The heatmap demonstrates the enriched GO terms in the virus- and mock-infected airway and nasal organoids. The color code represents the normalized enrichment score (NES) value for each enriched GO term.

We next performed bulk RNA sequencing analysis to investigate the host cellular response in the human airway and nasal organoids upon infection with H5N1-C or H5N1-VN1194. Transcriptomic analysis of human airway and nasal organoids revealed that both viruses triggered a strong cellular response in these organoids, including IFNs, ISGs, and proinflammatory cytokines and chemokines (Fig. 4a). The infections triggered more intense host responses in airway organoids than in nasal organoids, consistent with our prior observations in organoid infections with other respiratory viruses^17,20^. Gene Ontology (GO) enrichment analysis highlighted the significant pathways, including regulation of viral processes and innate immune response, in both airway and nasal organoids (Fig. 4b). Despite a less robust replication (Fig. 3), H5N1-VN1194 triggered a stronger cellular response than H5N1-C in nasal organoids, which might be a possible driver of higher pathogenicity of Clade 1 H5N1 infections seen in humans.

### Cross-neutralization of N1 antibodies against 2.3.4.4b H5N1 and ancestral H5N1 exclusively revealed in human airway organoids

The productive 2.3.4.4b H5N1 infection in human airway and nasal organoids stood in contrast to the real-world scenario in the U.S., where mild human infections have been detected in the context of widespread fatal disease in other mammal species^33^. A possible reason is that pre-existing cross-reactive antibodies in humans may provide some protection against 2.3.4.4b H5N1 infection. Since most individuals had limited exposure to H5N1 viruses^34^, sera from healthy donors have no significant neutralizing antibodies to H5 HA^2^. However, inhibitory antibodies to the 2.3.4.4b H5N1 NA detected by the enzyme-linked lectin assay are present in healthy human sera, with titers equivalent to those to (H1N1)pdm09^2,35–40^. NA is a multifunctional protein with an important role at many stages of the infectious process, including viral entry and release from infected cells^41^. NA-mediated cleavage of sialic acids enables virus movement through mucus, which is essential for influenza virus infection of human respiratory cells. Unlike well-established neutralization assays for measuring HA antibodies, no readily conductible assay is available that directly measures the neutralizing activity of NA antibodies, since commonly used cell lines lack such mucus on top of monolayers^42,43^. Goblet cells in airway organoids secrete mucin onto the apical side of the organoid monolayers (Supplementary Figs. 3 and 4), like the in vivo counterpart, which can secrete mucin to form a mucus blanket in native airway mucosa.

**Fig. 5:**
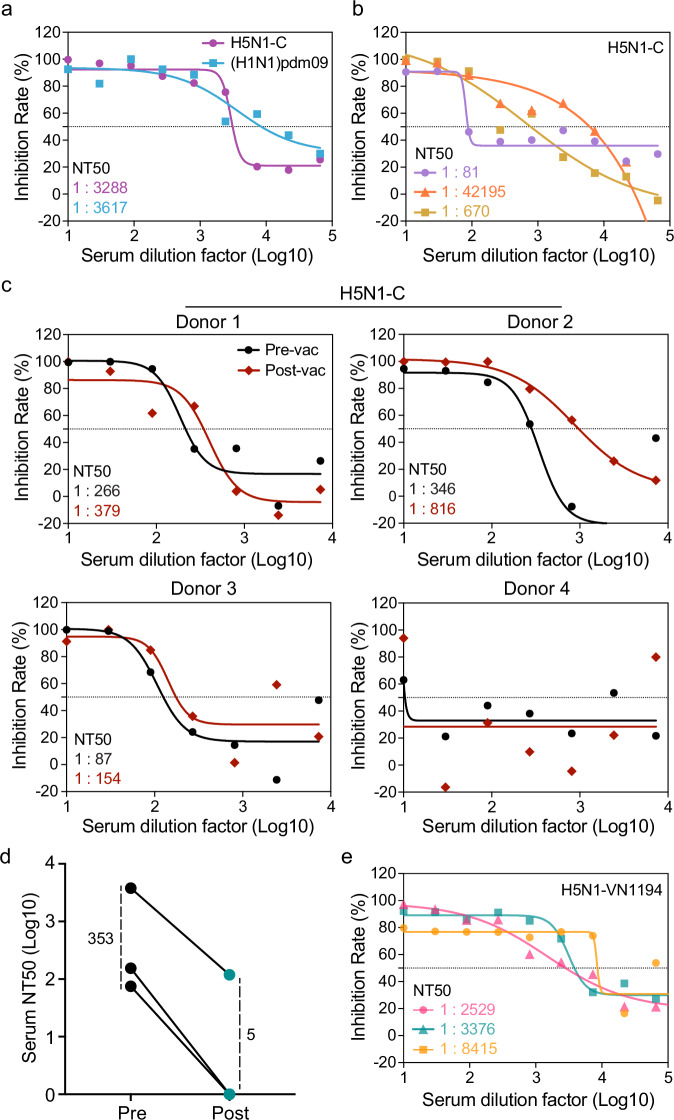
Cross-neutralization of human vaccination sera against 2.3.4.4b H5N1 in human airway organoids. **a** Neutralization activity of a vaccination serum against cattle H5N1 and (H1N1)pdm09 in human airway organoids. **b** Neutralization activity of three vaccination sera against cattle H5N1. **c** Neutralization efficacy of seasonal influenza pre-vaccinated (Pre-vac) and post-vaccinated (Post-vac) serum from four individual donors against cattle H5N1 virus. **d** The three vaccination sera neutralizing 2.3.4.4b H5N1 were applied to the depletion of (H1N1)pdm09 NA reactive antibodies. The three paired sera, i.e., pre-deleted (Pre) and deleted (Post), were subjected to the organoid-based neutralization assay against 2.3.4.4b H5N1. Numbers indicate geometric mean titre. **e** Neutralization activity of three vaccination sera against H5N1-VN1194. Data represent the mean from at least three replicates. Neutralization titers (NT50) were calculated by using a four-parameter logistic curve-fitting. Source data are provided as a Source data file.

Given the high biological relevance of human airway organoids to the native airway epithelium, including the formation of a mucus layer, we hypothesized that human airway organoids would be a favorable model to evaluate whether NA antibodies can neutralize influenza virus entry and protect human respiratory cells from infection. We measured the effect of human sera against H5N1-C infection, using mature human airway organoids grown on 96-transwell inserts, similar to conventional neutralization assays, in which MDCK cells are seeded in 96-well plates. First, we verified neutralization against (H1N1)pdm09 in MDCK cells using a serum specimen from a vaccinee who recently received the 2024/25 Quadrivalent influenza vaccine, containing A/(H1N1)pdm09, A/H3N2, and two influenza B antigens. As expected, the serum showed strong neutralization against (H1N1)pdm09 (NT50, 1:5652) in MDCK cells (Supplementary Fig. 5a). The parallel assay in human airway organoids revealed a similar neutralization of the serum against (H1N1)pdm09 (NT50 1:3617) infection (Fig. 5a). Interestingly, the serum neutralized H5N1-C with substantial potency (NT50 1:3288; Fig. 5a) in human airway organoids, but not in MDCK cells (Supplementary Fig. 5b). Additional serum specimens were collected from three other individuals after a recent seasonal influenza vaccination. All three serum specimens had neutralization activity against H5N1-C infection in human airway organoids, with NT50 readings of 1:81, 1:42195, and 1:670 (Fig. 5b).

Given the prevalence of cross-reactive antibodies to 2.3.4.4b H5N1 NA in humans^2^, we hypothesized that N1 antibodies induced by seasonal influenza vaccination and/or prior (H1N1)pdm09 infections may contribute to serum neutralization against H5N1-C in organoids. As such, we collected paired serum specimens from four vaccinees before and two weeks after seasonal influenza vaccination. In the MDCK cell-based assay, all four sera had neutralizing antibodies against the (H1N1)pdm09 virus, with increased neutralization titers post-vaccination (Supplementary Fig. 5c); while none of the four sera had cross-neutralization against H5N1-C (Supplementary Fig. 5d), consistent with a prior report^2^. In contrast, neutralizing antibodies against H5N1-C were present in the pre-vaccination sera from three out of four donors in the organoid-based assay, with around a 2-fold increase in neutralization titre after seasonal influenza vaccination (Fig. 5c).

To verify that serum antibodies against the NA of (H1N1)pdm09 were responsible for the observed neutralization against H5N1-C in human airway organoids, we pretreated the post-vaccination sera of the three positive donors (donors 1, 2, and 3 shown in Fig. 5c) with the recombinant (H1N1)pdm09 NA protein to deplete N1-specific antibodies. After verifying efficient antibody depletion (Supplementary Fig. 6), we then re-examined the neutralization activity of paired sera (i.e., sera before and after depletion) against H5N1-C. The cross-neutralization against H5N1-C of all three vaccination sera diminished dramatically after depleting the antibodies reactive with the (H1N1)pdm09 NA (Fig. 5d). We then inferred that these N1 antibodies may also neutralize H5N1-VN1194. As such, we examined the neutralization activity of the three post-vaccination sera against the ancestral H5N1-VN1194 in human airway organoids. We found that all three serum specimens also neutralized H5N1-VN1194 in human airway organoids (Fig. 5e).

Overall, we developed a novel organoid-based neutralization assay that directly measures the neutralizing potency of NA antibodies, thus overcoming a major limitation of conventional cell line-based neutralization assays. The results of the organoid-based neutralization assay suggested that a substantial proportion of the human population had cross-neutralizing N1 antibodies against H5N1 2.3.4.4b, and seasonal influenza vaccination enhanced the cross-neutralization capability. The antisera derived from seasonal influenza vaccination also neutralized the ancestral H5N1-VN1194. The prevalence of these cross-neutralizing N1 antibodies in the general population may have effectively protected the exposed individuals from 2.3.4.4b H5N1 and H5N1-VN1194 infection and ameliorated disease severity in the afflicted persons.

## Discussion

Since the first case of 2.3.4.4b H5N1 virus infection in dairy cows in the U.S. in 2024, the virus has spread to hundreds of farms in several states^6^ and spilled over to other mammals, including humans^9^. In this study, we developed cattle organoid models and human organoid-based assays to elucidate respiratory susceptibility to 2.3.4.4b H5N1 virus in cattle and humans and explore the impact of pre-existing cross-reactive NA antibodies in humans.

Building upon the techniques of deriving human respiratory and breast organoids^19,23,25,26^, we established organoid cultures of cattle airway epithelium and mammary glands. The derived cattle organoids were directed into an immature state, enabling a stable and long-term expansion. Upon induction of maturation, the differentiated cattle airway organoids contained abundant ciliated cells, as well as goblet cells, and club cells. More importantly, cattle airway organoids recapitulated cattle’s real-world susceptibility to influenza D virus^44^, which further elevated the biological relevance of these organoids.

Clade 2.3.4.4b H5N1 viruses are believed to be transmitted among cattle through contaminated milking equipment or a mouth-to-teat route, whereby the infectious virus is introduced into cattle mammary glands^45^. The robust replication of clade 2.3.4.4b H5N1 in mammary organoids recapitulated the mammary tropism seen in wild and experimental cattle^6^. Kim et al. recently reported 2.3.4.4b H5N1 infection of cattle mammary organoid, yet without detailed characterization to verify the biological relevance of the derived mammary organoids^46^. In addition, TPCK-trypsin was supplemented during virus inoculation, implying that the cattle mammary organoids per se may not sustain viral growth. Ex vivo udder explants were also used to demonstrate 2.3.4.4b H5N1 mammary infection^47^, yet these explants are not readily available to most research laboratories and have an inherent issue of tissue heterogeneity. The cattle organoid models reported in this study mitigate these limitations and variability, providing a more robust and reproducible platform. The active 2.3.4.4b H5N1 replication in cattle airway organoids recapitulated cattle respiratory infection upon intranasal inoculation or respiratory aerosol inoculation^10,11^. More importantly, the results suggest that underrecognized respiratory transmission may be contributing to the outbreaks. The enhanced replication fitness of 2.3.4.4b H5N1 relative to the ancestral H5N1-VN1194 might be related to accumulated mutations, especially in PB2^47,48^. Specifically, the novel reassortant B3.13 genotype acquired the PB2-M631L substitution to enhance interaction with bovine ANP32 proteins^48^. This key adaptation optimizes viral polymerase activity, driving efficient replication in bovine and other mammalian hosts, including humans^48^.

Despite the widespread nature of 2.3.4.4b H5N1 viruses in cattle and other mammals, clinically confirmed human infections remain comparatively limited, mostly presenting with mild symptoms^7,8^, although the low incidence might be attributed to under-reporting^49^. Nonetheless, the high replication of 2.3.4.4b H5N1 in human airway and nasal organoids appeared to contradict the mild disease in human infections. In addition, the puzzling discrepancy between rare and mild human infection cases in the U.S. versus the severe and fatal infection in ferrets, the animal model relevant to human infection, raised the possibility of pre-existing immunity in humans. Sidney et al reported that pre-existing T cell cross-reactivity against seasonal H1N1 viruses may blunt the severity of human H5N1 infection^50^. Matthew et al also demonstrated that humoral immunity elicited by N1 can partially protect against H5N1 infection in mice^36^. Pre-existing cross-reactive antibodies targeting the 2.3.4.4b NA have been identified in healthy human sera based on the enzyme-linked lectin assay^2,38–40^. Several recent studies further demonstrated that prior infection with (H1N1)pdm09 induced cross-reactive antibodies to 2.3.4.4b H5N1 viruses and reduced the susceptibility and disease severity in ferrets^51–53^. However, no readily conductible assay is available that can directly measure the activity of NA antibodies to neutralize influenza virus entry. In standard neutralization assays, immortalized cells, e.g., MDCK cells, are inoculated with viruses pre-incubated with serially diluted antibodies or antisera. The setting only measures the activity of neutralizing antibodies targeting HA; the neutralizing activity of NA antibodies cannot be manifested since NA-mediated penetration of the mucus blanket^42^, a critical step for infecting target cells in the respiratory epithelium, does not occur in MDCK cells. Goblet cells in the mature nasal and airway organoids secrete abundant mucins onto the organoid monolayers, thus providing a unique and biologically relevant model to demonstrate the essential role of NA for penetrating mucus and facilitating virus infection of human respiratory cells.

In this study, we developed an original organoid-based neutralization assay and demonstrated NA antibody-mediated neutralization against 2.3.4.4b H5N1 and H5N1-1194 (Fig. 5), which was not detectable in the cell line-based neutralization assay performed in parallel. Through the organoid-based neutralization assay, we found that a substantial proportion of normal human sera had cross neutralizing antibodies against 2.3.4.4b H5N1, suggesting that pre-existing N1 immunity offers cross-protection; and seasonal influenza vaccination enhanced the cross-neutralization against 2.3.4.4b H5N1 and ancestral H5N1-1194. Of note, the antibody depletion experiment explicitly evidenced that cross-neutralization against 2.3.4.4b H5N1 was largely conferred by antibodies targeting (H1N1)pdm09 NA (Fig. 5d).

Despite the high infectivity of 2.3.4.4b H5N1 in human airway and nasal organoids, pre-existing cross-reactive N1 antibodies in population sera cross-protect human respiratory cells from 2.3.4.4b H5N1 infection. This, together with the weakened innate immune response triggered by 2.3.4.4b H5N1 compared to H5N1-1194, may constitute the cellular mechanisms underlying the mild disease in most 2.3.4.4b H5N1 human infections. Thus, human respiratory organoids can simultaneously examine viral infectivity, host response, and humoral immune protection, enabling an overall assessment of human susceptibility and viral pathogenicity in a biologically relevant setting. Yet, as we only tested a limited number of human sera in this study, the role of NA antibody-mediated humoral immunity against 2.3.4.4b H5N1 and H5N1-1194 in the general population warrants a large-scale investigation by testing more human serum specimens. Moreover, as qPCR-based quantification of viral load was used to measure viral replication and antibody-mediated neutralization, the resulting NT50 values are likely higher than those obtained from conventional cell-line-based neutralization assays, where NT50 is commonly determined by viral titration. Nonetheless, the results of the organoid-based assay clearly demonstrate the presence of N1-neutralizing antibodies in the sera of a proportion of healthy individuals. As the neutralization assay here relies on respiratory organoid monolayers cultured on transwell inserts, more accurate virus quantification methods tailored to this platform, such as focus-forming assays coupled with advanced imaging systems, should be developed to measure readouts from organoid-based neutralization assays more precisely.

Collectively, our findings of the high replicative fitness of 2.3.4.4b H5N1 in cattle mammary and airway organoids revealed the viral trait driving the expanding outbreaks of 2.3.4.4b H5N1 viruses. The underrecognized respiratory transmission may be contributing to the outbreaks. More importantly, the organoid-based neutralization assay provides a transformative and accurate evaluation of NA antibodies in a biologically relevant setting. Human serum neutralization induced by prior influenza infections and whole virus-based vaccination derives from the combined protection from HA and NA antibodies. The novel organoid-based neutralization assay unprecedentedly enables the measurement of the combined neutralization activity from both types of antibodies. Overall, human respiratory organoids offer an unprecedented and realistic platform for assessing population humoral immunity and vaccination effectiveness, evaluating human susceptibility to respiratory pathogens, and guiding public health preparedness.

## Methods

### Establishment, maintenance, and differentiation of organoids

Human lung tissue-derived organoids and nasal organoids were established and expanded using a respiratory organoid expansion medium (BiomOrgan, A01-001), in accordance with ethical approval from the Institutional Review Board at the University of Hong Kong/Hospital Authority Hong Kong West Cluster (UW21-695)^17,24,54^. Informed consent was obtained from patients and volunteers for obtaining human lung tissues and nasal cells. The derived organoids were passaged every 10–14 days at ratios ranging from 1:3 to 1:10 using the same expansion medium. Proximal differentiation to generate monolayers of mature human airway organoid (hAwO) and human nasal organoid (hNsO) on transwell inserts has been previously described^18,19^. Briefly, undifferentiated organoids were dissociated into single cells using 10X TrypLE Select (ThermoFisher, A1217701) for 5 min at 37 °C and seeded onto Transwell inserts at a density of 1.5 × 10⁵ cells per insert. The cells were cultured in the expansion medium for 2 days, then switched to the proximal differentiation (PD) medium and incubated for 14 days.

Cattle lung tissue-derived organoids and mammary organoids were established following the culture protocol for human lung tissue-derived organoids^19^ and human breast organoids^25^, respectively. Briefly, small pieces of lung and mammary gland tissues (approximately 0.2–0.6 cm²) were collected from a 2-year-old lactating Holstein–Friesian cow. Tissues were minced and digested with 2 mg/mL collagenase (Sigma-Aldrich, C9407) for 1–2 h at 37 °C, followed by shearing using a glass Pasteur pipette (BRAND, 747720) and filtration through a 100 μm cell strainer (Corning, 352360). Single cells were then embedded in 70% Matrigel (Corning, 356231) and dispersed into 24-well suspension culture plates. After the Matrigel was solidified, the droplets of cattle lung cells were incubated with a respiratory organoid expansion medium (BiomOrgan, A01-001) at 37 °C in a humidified incubator with 5% CO₂. The derived organoids were passaged every one to two weeks. Monolayers of differentiated cattle airway organoids were generated based on the proximal differentiation protocols for human airway organoids as described previously^19^. In this study, monolayers of differentiated human airway organoids, human nasal organoids, and cattle airway organoids were used for the experiments throughout. Cattle mammary organoids were cultured and expanded with culture medium supplemented with growth factors^26^ (Supplementary Table 1). The expanding mammary organoids were cultured with medium without EGF for two weeks to enrich the luminal cells according to the protocol described previously^26^.

### Virus infection and detection

Recombinant cattle H5N1 virus (A/dairy cattle/Texas/24-008749-003-origin/2024), H5N1 VN1194 (A/Vietnam/1194/04), and H1N1 pdm09 (A/Hong Kong/415742/2009) were plaque-purified and subsequently propagated in MDCK cells obtained from ATCC. For the infection of airway and nasal organoids, media in the top and bottom chambers were first removed. After washing once with a basal medium (Advanced DMEM/F-12 (Gibco), 1% HEPES, 1% GlutaMAX, and 1% Penicillin/Streptomycin), the organoids were inoculated with the indicated viruses at the indicated MOI for 2 h, followed by incubation in the basal medium at 37 °C^17,24^. The 3D cattle mammary organoids were sheared mechanically to expose the apical surface to the virus inoculum. To accurately determine the MOI for virus inoculation, organoids in one Matrigel droplet were enzymatically dissociated into single cells, and the total cell number was quantified prior to the infection assays. The sheared organoids (approximately 2 × 10^5^ cells) were then incubated with the indicated viruses at the indicated MOI for 2 h at 37 °C in a 24-well suspension plate. After washing with basal medium, the inoculated organoids were suspension-cultured in the basal medium.

To evaluate viral replication kinetics, culture supernatants were collected at the indicated time points post-infection, and viral RNA was extracted using the QIAamp Viral RNA Mini Kit (QIAGEN, 52904). Viral loads were quantified by RT-qPCR targeting the viral M gene, and infectious titers were determined by TCID50 assay and/or plaque assay as previously described^19^. All experiments involving the cattle H5N1 virus and H5N1 VN1194 virus were conducted in Biosafety Level 3 laboratories upon approval by the Faculty of Medicine, The University of Hong Kong.

### Immunofluorescence staining and confocal imaging

Virus- or Mock-infected organoids were applied to confocal imaging after immunostaining as described previously^17,18,22^. Briefly, the fixed organoids were permeabilized with 0.5% Triton X-100 for 10 min and blocked with 3% BSA/PBS for 1 h, followed by incubation with primary antibodies and secondary antibodies (Supplementary Table 2) as described previously^19^. Nuclei and actin filaments were counterstained with DAPI (ThermoFisher, D21490) and Phalloidin-Atto 647 (Sigma-Aldrich), respectively. After staining, the organoids were whole-mounted on a glass slide with ProLong™ Glass Antifade Mountant (ThermoFisher, P36580). Confocal images were acquired using a Carl Zeiss LSM 980 confocal microscope. Image processing was performed using the ZEN blue software.

### Flow cytometry analysis

Virus- or Mock-infected organoids were dissociated into single cells with 10 mM EDTA (ThermoFisher, AM9260G) for 30–60 min at 37 °C. The cells were then fixed with 4% PFA and permeabilized with 0.1% Triton X-100, followed by immunostaining using an influenza A nucleoprotein (NP) antibody (Abcam, ab128193) and a secondary antibody as previously described^17,18^. Flow cytometry analysis was performed using an Agilent Novocyte Advanteon BVR. Data processing was conducted with FlowJo 10.4.0 software.

### Neutralization assay in human airway organoids and MDCK cells

Human sera were collected upon ethical approval from the Institutional Review Board at the University of Hong Kong/Hospital Authority Hong Kong West Cluster (UW24-279). Informed consent was obtained from patients and volunteers prior to serum collection. For neutralization assays in human airway organoids, media in the top and bottom chambers were first removed. The apical and basolateral sides were washed once with the basal medium, which does not remove the mucus layer on the apical side. Subsequently, 150 μl of medium was added in the bottom chamber of each well. H5N1-C (0.01 MOI), (H1N1)pdm09 (0.01 MOI), and H5N1-VN1194 (0.01 MOI) were mixed with serially diluted human sera in 96-well plates. Each serum sample was prepared using 10 serial 3-fold dilutions, ranging from an initial dilution of 1:10 down to 1:196,830. After incubation at 37 °C for 1 h, 50 μl of the virus-serum mixture was transferred to the top chamber in 96-well transwell inserts seeded with human airway organoids, or MDCK cells seeded in 96-well plates and incubated at 37 °C for an additional 2 h. After washing twice with the basal medium, the infected organoids were maintained with basal medium in top and bottom chambers. At 24 h post-infection, 50 μl of supernatant was collected from each well and mixed with AVL lysis buffer. RNA was extracted using the RNeasy Pure mRNA Bead Kit (QIAGEN, 931636). Viral loads were quantified via RT-qPCR analysis. RT-qPCR Ct values were converted to an inhibition rate using the 2^−ΔCt^ method. The inhibition rate was calculated as follows: ($1-2^{(-{({{\rm{Ct}}_{\rm{sample}}}-{\rm{Ct}}_{{\rm{virus}} \, {\rm{only}} \, {\rm{control}}})})}$) × 100%, where Ct_virus only control_ represents the mean Ct value from at least three replicates of virus-only wells. Upon the transformation, NT50 values were determined using a 4-parameter non-linear regression analysis in GraphPad Prism 9 (GraphPad Software).

### NA antibody depletion

The recombinant H1N1(pdm09) NA proteins (A/Brisbane/02/2018 (H1N1)pdm09) were biotinylated with EZ-Link NHS-PEG4-Biotin (ThermoFisher, A39259) for 2 h on ice. Excess unbound biotin was removed by passing the mixture through Zeba spin desalting columns (ThermoFisher). Streptavidin magnetic beads (ThermoFisher, 88817) were washed twice with PBST and incubated with the biotinylated recombinant NA proteins at a ratio of 30 μg per mg of beads in PBS for 30 min at room temperature on a rotating mixer. Subsequently, the beads were washed twice with PBS and then incubated overnight at 4 °C with human serum samples, with gentle agitation. After incubation, a magnet will be used to separate the beads from the supernatant, which contains the non-NA-binding antibodies. The depleted serum specimen was transferred to a new tube and stored at 4 °C for subsequent neutralization assays. The depletion efficiency was assessed by a sandwich ELISA.

### Transmission electron microscopy

Cattle mammary organoids were embedded in resin after sequential fixation in 2.5% glutaraldehyde and 1% osmium. The ultrathin sections were stained with uranyl acetate and examined under a Philips CM 100 transmission electron microscope.

### RNA sequencing analysis

Differential expression analysis was performed using DESeq2. Genes with log2|fold change| > 1 and adjusted *p*-value < 0.05 were considered significantly different. Heatmaps of gene expression levels were constructed using the Pheatmap R package (https://cran.r-project.org/web/packages/pheatmap/index.html). Gene set enrichment analysis (GSEA) was performed using the fgsea R package (https://github.com/ctlab/fgsea). Analysis and visualization of RNA-seq data were carried out in R v4.3.2. Heatmaps of comparison and pathway were created by the ComplexHeatmap v2.18.0 R package. The RNA-Seq data were uploaded to NCBI Gene Expression Omnibus (GEO) the accession number GSE306043 (token ybgxmcqynhefhyh).

### Statistical analysis

Statistical analysis was conducted using GraphPad Prism 9.0. Student’s *t*-test was used to determine statistical significance as specified in the Fig. legends. The number of replicates is indicated in the figure legends. *P ≤ 0.05, **P ≤ 0.01, ***P ≤ 0.001.

### Reporting summary

Further information on research design is available in the Nature Portfolio Reporting Summary linked to this article.

## Supplementary information

Supplementary Information

Reporting summary

Transparent Peer Review file

## Source data

Source data

## Data Availability

The RNA-seq data generated in this study have been deposited in the GEO database under the accession code GSE306043. Source data are provided in this paper. Source data are provided with this paper.
