# Supplementary material for: Cattle and human organoids reveal 2.3.4.4b H5N1 cross-species transmission potential and neuraminidase-specific neutralizing antibodies in humans
Source: Nat Commun. 2026 Jul 8;17:5585. doi: 10.1038/s41467-026-74345-w (PMC13346980; doi:10.1038/s41467-026-74345-w)
Supplement: Supplementary file 1 — Supplementary Information [file 41467_2026_74345_MOESM1_ESM.pdf]

Supplementary Fig. 1. The replication kinetics of (H1N1)pdm09 in human airway organoids.

a, b Human airway organoids derived from two donors were inoculated with (H1N1)pdm09 at 0.01 MOI (n=3). Culture media were harvested from the organoids at the indicated time points for viral load detection and viral titration by plaque assay. Data represent mean and SD of the indicated number (n) of biological replicates. Source data are provided as a Source Data file.

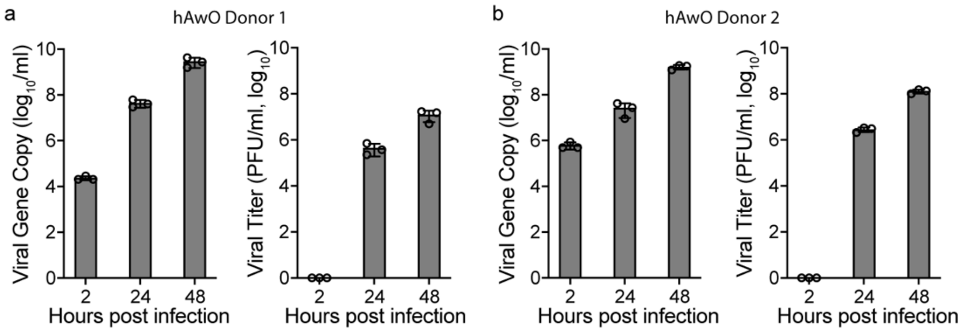

Supplementary Fig. 2. Gating strategy for the flow cytometry experiment in Fig. 2c, 3e, 3f.

The image illustrates the gating strategy employed for analyzing 2.3.4.4b H5N1, H5N1 VN1194 and H1N1pdm NP+ cells within infected organoids.

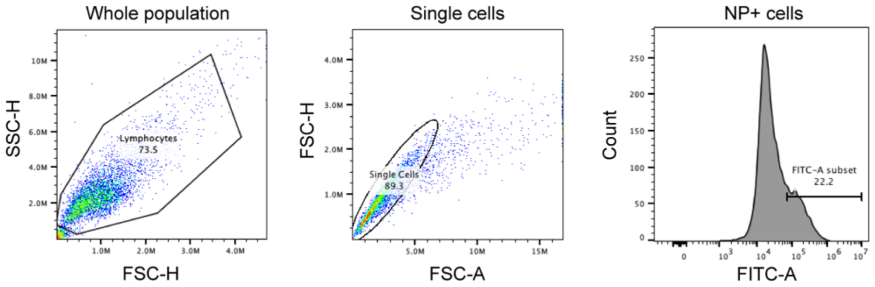

Supplementary Fig. 3. Confocal imaging of MUC5AC+ goblet cells in human airway organoids.

Confocal images of MUC5AC+ goblet cells (green) and ACCTUB+ ciliated cells (red) in human airway organoids. Nuclei and actin filaments were counterstained with DAPI (blue) and Phalloidin-647 (white), respectively. The experiment was independently repeated three times with similar results. Scale bar, 10 μm.

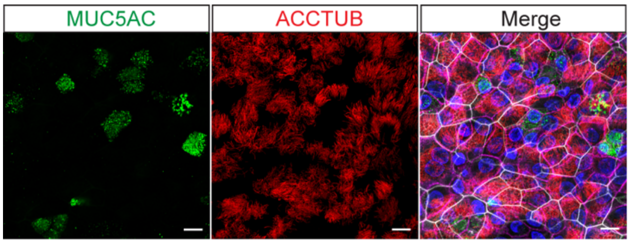

Supplementary Fig. 4. Formation of a mucus layer on the top of human airway organoids.

Periodic acid-Shiff (PAS) staining of human airway organoid monolayers differentiated in transwell insert. **a** En face images of the organoid monolayer (left) and the monolayer after mucus depletion (right). Scale bar, 20 μm. **b** Cross-section images show the mucus layer on top of the organoid monolayer. The experiment was independently repeated three times with similar results. Scale bar, 20 μm.

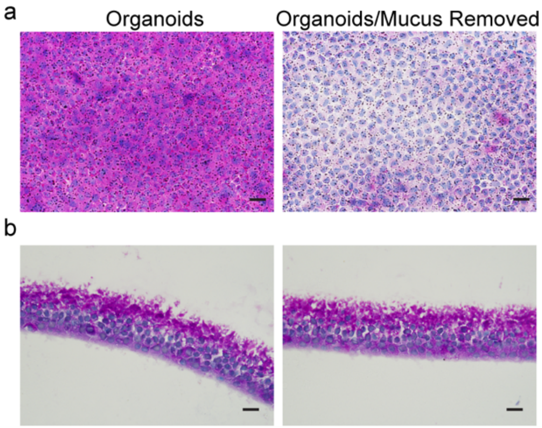

Supplementary Fig. 5. Neutralization activity of vaccination sera against 2.3.4.4b H5N1 and (H1N1)pdm09 in MDCK cells.

a, b Neutralization activity of a vaccinee serum against (H1N1)pdm09 (**a**) and 2.3.4.4b H5N1 (**b**) virus in MDCK cells. **c** Neutralization potency of pre-vaccination (Pre-vac) and post-vaccination (Post-vac) serum from four donors against (H1N1)pdm09 in MDCK cells. **d** Neutralization activity of pre- and post-vaccination sera from the four donors against cattle H5N1 virus in MDCK cells. Data represent the mean from at least three replicates. NT50 was calculated by using a four-parameter logistic curve-fitting. Source data are provided as a Source Data file.

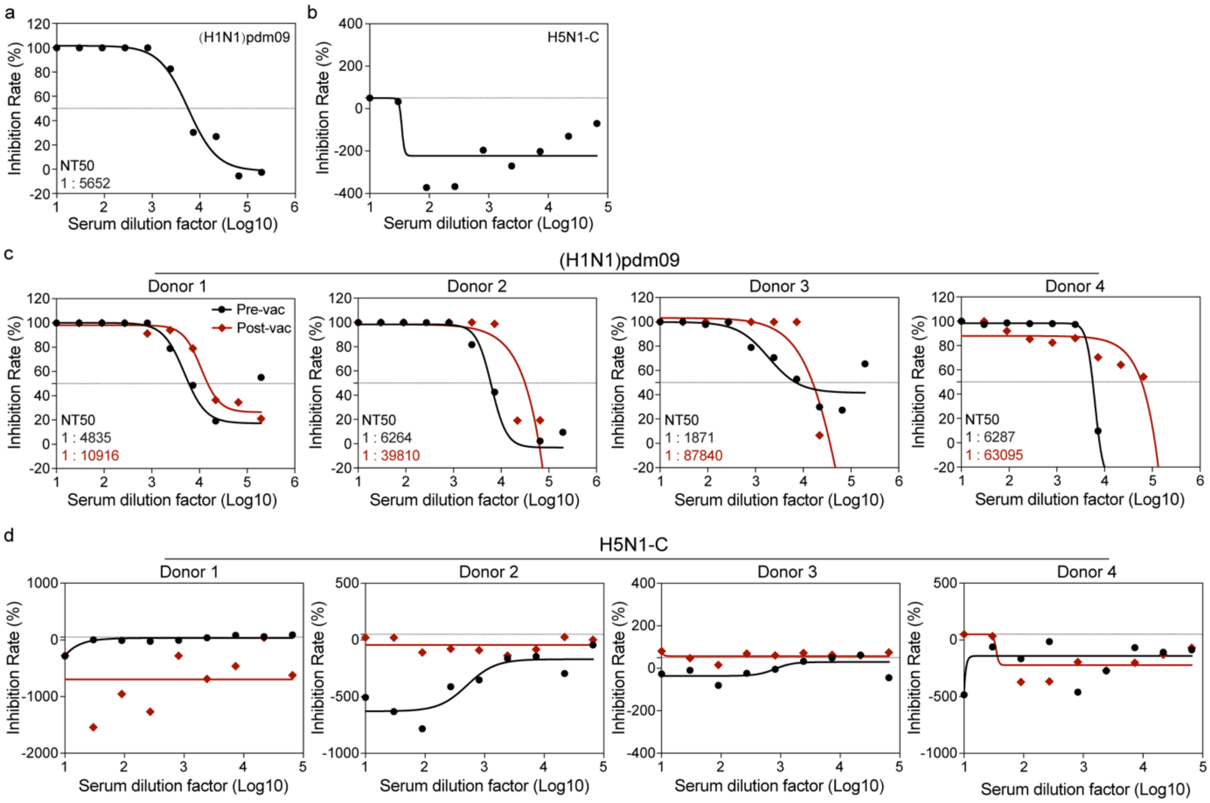

Supplementary Fig. 6. Level of NA reactive antibodies in three serum specimens before (Pre) and after depletion (Post).

Three post-vaccination serum specimens that showed neutralizing antibodies against cattle H5N1 in organoid-based neutralization were applied to the depletion of N1 NA reactive antibodies. Sandwich ELISA was performed to detect NA-reactive antibodies in serum specimens before (Pre) and after (Post) antibody depletion. Source data are provided as a Source Data file.

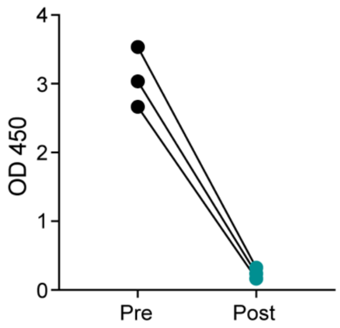

Supplementary Table 1. Composition of cattle mammary organoid culture medium.

**Reagents**	**Company**	**Catalog No.**	**Working concentration**
Advanced DMEM/F12	ThermoFisher	12634010	n/a
GlutaMAX (glutamine)	ThermoFisher	35050061	1X
HEPES 1M	ThermoFisher	15630-056	10mM
Penicillin-Streptomycin (10,000 U/mL)	ThermoFisher	15140-122	1X
Primocin	Invivogen	ant-pm-1	100μg/ml
N-Acetylcysteine	Sigma-Aldrich	A9165	1.25mM
Nicotinamide	Sigma-Aldrich	N0636	10mM
B27 supplement	ThermoFisher	17504-044	1X
Rspondin1	BiomOrgan	A04-001	500ng/ml
Noggin (conditioned medium)	BiomOrgan	A04-002	100ng/ml
FGF-10	BiomOrgan	A04-005	20ng/ml
FGF-7	BiomOrgan	A04-004	5ng/ml
EGF	BiomOrgan	A04-003	5ng/ml
Heregulin β-1	Peprotech	100-03	5nM
A8301	Tocris	2939	500nM
SB202190	Sigma-Aldrich	S7067	1μM
Y-27632	Tocris	1254	5μM

*Conditioned media were produced from stable cell lines for production of R-spondin1 and Noggin.

Supplementary Table 2. List of antibodies.

Antibody	Company	Catalog No.	Clone	Lot No.	Dilution
Anti-Uteroglobin/CC10 antibody	Proteintech	10490-1-AP	N/A	00115085	1:200
Anti-β-Tubulin IV antibody	Sigma	T7941	ONS.1A6	334324	1:500
Anti-beta IV Tubulin antibody	Abcam	ab179509	EPR16776	GR3438775-6	1:500
Anti-Mucin 5AC antibody	RayBiotech	188-10578-2	N/A	2733240418RAY8	1:200
Anti-Influenza A Virus Nucleoprotein antibody	Abcam	ab128193	C43	1133017-1	1:200
Anti-Mouse Alexa Fluor 488	Invitrogen	A11001	N/A	3268352	1:300
Anti-Mouse Alexa Fluor 594	Invitrogen	A11005	N/A	2765671	1:300
Anti-Rabbit Alexa Fluor 488	Invitrogen	A11008	N/A	3148302	1:300
Anti-Rabbit Alexa Fluor 594	Invitrogen	A11012	N/A	2616076	1:300
