# Supplementary material for: Cattle and human organoids reveal 2.3.4.4b H5N1 cross-species transmission potential and neuraminidase-specific neutralizing antibodies in humans
Source: Nat Commun. 2026 Jul 8;17:5585. doi: 10.1038/s41467-026-74345-w (PMC13346980; doi:10.1038/s41467-026-74345-w)
Supplement: Supplementary file 2 — Reporting summary [file 41467_2026_74345_MOESM2_ESM.pdf]

nature portfolio

Corresponding author(s): Jie ZHOU

Last updated by author(s): May 28, 2026

Reporting Summary

Nature Portfolio wishes to improve the reproducibility of the work that we publish. This form provides structure for consistency and transparency in reporting. For further information on Nature Portfolio policies, see our Editorial Policies and the Editorial Policy Checklist.

Statistics

For all statistical analyses, confirm that the following items are present in the figure legend, table legend, main text, or Methods section.

n/a	Confirmed	
☐	☒	The exact sample size (*n*) for each experimental group/condition, given as a discrete number and unit of measurement
☐	☒	A statement on whether measurements were taken from distinct samples or whether the same sample was measured repeatedly
☐	☒	The statistical test(s) used AND whether they are one- or two-sided<br>*Only common tests should be described solely by name; describe more complex techniques in the Methods section.*
☒	☐	A description of all covariates tested
☒	☐	A description of any assumptions or corrections, such as tests of normality and adjustment for multiple comparisons
☐	☒	A full description of the statistical parameters including central tendency (e.g. means) or other basic estimates (e.g. regression coefficient) AND variation (e.g. standard deviation) or associated estimates of uncertainty (e.g. confidence intervals)
☐	☒	For null hypothesis testing, the test statistic (e.g. *F*, *t*, *r*) with confidence intervals, effect sizes, degrees of freedom and *P* value noted<br>*Give P values as exact values whenever suitable.*
☒	☐	For Bayesian analysis, information on the choice of priors and Markov chain Monte Carlo settings
☒	☐	For hierarchical and complex designs, identification of the appropriate level for tests and full reporting of outcomes
☒	☐	Estimates of effect sizes (e.g. Cohen's *d*, Pearson's *r*), indicating how they were calculated

Our web collection on statistics for biologists contains articles on many of the points above.

Software and code

Policy information about availability of computer code

Data collection	No software is used.
Data analysis	GraphPad Prism 9.0 was used for data analysis and plotting the figures.<br>FlowJo software 10.4.0 was used for the analysis of flow cytometry data.<br>Heatmaps of gene expression levels were constructed using the Pheatmap R package (https://cran.r-project.org/web/packages/pheatmap/index.html).<br>Gene set enrichment analysis (GSEA) was performed using the fgsea R package (https://github.com/ctlab/fgsea).<br>Analysis and visualization of RNA-seq data were carried out in R v4.3.2.<br>Heatmaps of comparison and pathway were created by the ComplexHeatmap v2.18.0 R package.

For manuscripts utilizing custom algorithms or software that are central to the research but not yet described in published literature, software must be made available to editors and reviewers. We strongly encourage code deposition in a community repository (e.g. GitHub). See the Nature Portfolio guidelines for submitting code & software for further information.

Data

Policy information about availability of data

All manuscripts must include a data availability statement. This statement should provide the following information, where applicable:

- Accession codes, unique identifiers, or web links for publicly available datasets
- A description of any restrictions on data availability
- For clinical datasets or third party data, please ensure that the statement adheres to our policy

The RNA-seq data generated in this study have been deposited in the GEO database under the accession code GSE306043. Source data are provided in this paper.

Research involving human participants, their data, or biological material

Policy information about studies with human participants or human data. See also policy information about sex, gender (identity/presentation), and sexual orientation and race, ethnicity and racism.

Reporting on sex and gender	N/A
Reporting on race, ethnicity, or other socially relevant groupings	N/A
Population characteristics	N/A
Recruitment	N/A
Ethics oversight	Institutional Review Board of the University of Hong Kong/Hospital Authority Hong Kong West Cluster (UW24-279 and UW21-695). Informed consent was obtained from patients and volunteers for obtaining human lung tissue, nasal cells and serum.

Note that full information on the approval of the study protocol must also be provided in the manuscript.

Field-specific reporting

Please select the one below that is the best fit for your research. If you are not sure, read the appropriate sections before making your selection.

☒ Life sciences ☐ Behavioural & social sciences ☐ Ecological, evolutionary & environmental sciences

For a reference copy of the document with all sections, see nature.com/documents/nr-reporting-summary-flat.pdf

Life sciences study design

All studies must disclose on these points even when the disclosure is negative.

Sample size	No experiment presented in this study requires caculating sample size .
Data exclusions	No excluded data.
Replication	All infection experiments were replicated at least twice independently. Electron microscopy was performed once, resulting in at least five images. The multiplex PCR array was performed once in triplicate.
Randomization	Not applicable.
Blinding	Blinding is not required.

Reporting for specific materials, systems and methods

We require information from authors about some types of materials, experimental systems and methods used in many studies. Here, indicate whether each material, system or method listed is relevant to your study. If you are not sure if a list item applies to your research, read the appropriate section before selecting a response.

Materials & experimental systems

n/a	Involved in the study
☐	☒ Antibodies
☐	☒ Eukaryotic cell lines
☒	☐ Palaeontology and archaeology
☒	☐ Animals and other organisms
☒	☐ Clinical data
☒	☐ Dual use research of concern
☒	☐ Plants

Methods

n/a	Involved in the study
☒	☐ ChIP-seq
☐	☒ Flow cytometry
☒	☐ MRI-based neuroimaging

Antibodies

Antibodies used

Antibody	Company	Catalog No.	Clone	Lot No.	Dilution
Anti-Uteroglobin/CC10 antibody	Proteintech	10490-1-AP	N/A	00115085	1:200
Anti-β-Tubulin IV antibody	Sigma	T7941	ONS.1A6	334324	1:500
Anti-beta IV Tubulin antibody	Abcam	ab179509	EPR16776	GR3438775-6	1:500
Anti-Mucin 5AC antibody	RayBiotech	188-10578-2	N/A	2733240418RAY8	1:200
Anti-Influenza A Nucleoprotein antibody	Abcam	ab128193	C43	1133017-1	1:200
Anti-Mouse Alexa Fluor 488	Invitrogen	A11001	N/A	3268352	1:300
Anti-Mouse Alexa Fluor 594	Invitrogen	A11005	N/A	2765671	1:300
Anti-Rabbit Alexa Fluor 488	Invitrogen	A11008	N/A	3148302	1:300
Anti-Rabbit Alexa Fluor 594	Invitrogen	A11012	N/A	2616076	1:300

Validation

The application of primary antibodies for immunostaining has been validated by the providers.

Eukaryotic cell lines

Policy information about cell lines and Sex and Gender in Research

Cell line source(s)

MDCK cell line is purchased from ATCC.
Cattle airway and mammary organoids are established from the lung and mammary gland tissues collected from a 2-year-old lactating Holstein-Friesian cow.
Human airway and nasal organoids are established from human lung tissue and nasal epithelial cells.

Authentication

MDCK cells were not authenticated.
Cattle airway organoids are authenticated with immunofluorescence staining.
Cattle mammary organoids are authenticated with TEM imaging.
Human airway and nasal organoids are authenticated with immunofluorescence staining and RT-qPCR analysis.

Mycoplasma contamination

MDCK, cattle and human organoids tested negative for mycoplasma contamination.

Commonly misidentified lines
(See ICLAC register)

No commonly misidentified lines were used.

Plants

Seed stocks

No seed was used in this study.

Novel plant genotypes

No plants were used in this study.

Authentication

No plants were used in this study.

Flow Cytometry

Plots

Confirm that:

☒ The axis labels state the marker and fluorochrome used (e.g. CD4-FITC).

☒ The axis scales are clearly visible. Include numbers along axes only for bottom left plot of group (a 'group' is an analysis of identical markers).

☒ All plots are contour plots with outliers or pseudocolor plots.

☒ A numerical value for number of cells or percentage (with statistics) is provided.

Methodology

Sample preparation	Organoids were dissociated into single cells with 10mM EDTA at 37°C for 30-60 minutes and fixed with 4% PFA for 30 minutes at room temperature. Cells were permeabilized with 0.1% Triton X-100 for 5 minutes at 4°C, and stained with primary and secondary antibodies.
Instrument	Agilent NovoCyte Quanteon analyzer
Software	FlowJo software Version: 10.7.1 is used for the analysis of flow cytometry data. GraphPad Prism 9.0 is used for data analysis.
Cell population abundance	The cells were not sorted in our flow cytometry analysis.
Gating strategy	1) FSC-A and SSC-A were used to gate for the bulk population of cells, 2) FSC-H and FSC-W or SSC-H and SSC-W were used to gate for the single cells, 3) NP positive populations were gated compared to Mock-infected cells.

☒ Tick this box to confirm that a figure exemplifying the gating strategy is provided in the Supplementary Information.
