# Supplementary material for: Cattle and human organoids reveal 2.3.4.4b H5N1 cross-species transmission potential and neuraminidase-specific neutralizing antibodies in humans
Source: Nat Commun. 2026 Jul 8;17:5585. doi: 10.1038/s41467-026-74345-w (PMC13346980; doi:10.1038/s41467-026-74345-w)
Supplement: Supplementary file 3 — Transparent Peer Review file [file 41467_2026_74345_MOESM3_ESM.pdf]

nature portfolio

Peer Review File

Cattle and human organoids reveal 2.3.4.4b H5N1 cross-species transmission potential and neuraminidase-specific neutralizing antibodies in humans

Corresponding Author: Professor Jie Zhou

This file contains all reviewer reports in order by version, followed by all author rebuttals in order by version.

Version 0:

Reviewer comments:

Reviewer #1

(Remarks to the Author)
The article titled "Cattle and human organoids reveal cross-species transmission potential of 2.3.4.4b H5N1 and neuraminidase-specific neutralizing antibodies in humans" is an experimental study aimed at developing and testing cattle airway and mammary organoids against HPAI 2.3.4.4b H5N1 virus in comparison with human airway and nasal organoid susceptibility. Additionally, the authors examined the impact of pre-existing cross-reactive NA antibodies in human organoids neutralization assays with 2.3.4.4b virus.

The article is well written and contains novel and impactful information regarding influenza virus replication and cell tropism. The reviewer has a few minor revisions that would benefit the manuscript:
1) Starting from the title and throughout the manuscript, the authors mention concepts such as "transmission", "protection from infection", etc. these are claims that the authors cannot prove with just the experiments listed within the manuscript. I would revise the authors' title and conclusions.
2) The methods cited for the generation of the cattle airway and mammary gland organoids are not specific for cattle. Given the radically different anatomy between human and cattle, and the fact that both the mammary gland and airways cell types, receptor distribution and structures vary considerably depending on the collection site, production/not in production stage, age of the animal, etc. I would strongly recommend adding those specific details to the materials and methods of manuscript.
3) After EGF modifications, the CMO microscopically appear rather to be inverted (epithelial lining on the outer side) while also maintaining some minimal intra-luminal invaginations lined by squamous epithelium. This appears evident in both Figure 1F and 2E. Please add these details to the manuscript.
4) At histology, the cattle mammary alveoli are involuted (not secreting) while at EM of the CMOs the authors sustain that they see milk production. Please explain this discrepancy.
5) Given that the organoids were developed from inactive mammary glands, while most the outbreak were from lactating cattles, could the authors comment on the possibility to develop fully active/secreting mammary alveoli and whether milk production would impact virus replication.
6) Line 95 – Cuboidal cells lining the alveoli of the mammary gland are intrinsically different from the cells lining the ducts and secretory portions. Alveolar cells are secretory while ductular cells are not. Please revisit.
7) It would be interesting to confirm whether the high replication in organoids can be explained by the proportion and cell types present in culture, and whether that differs significantly from the original in vivo sample. Given that IF markers for cattle are limited, my recommendation is to perform single cell sequencing analysis. This will allow proper characterization of the cell types present, their transcriptomic profiles and their frequencies.
8) The reviewer suggests the use of Alcian blue histochemistry and/or IF for the specific visualization of mucins. PAS is less specific and overlaps with cytoplasmic polysaccharides and glycoproteins/lipids. Mucin IF, instead is more specific for mucin glycoproteins and various markers are available (e.g., MUC5AC, MUC5B, MUC2).
9) Please explain why in the VN, viral loads were assessed by PCR instead of HA or virus titration? Also, most microneutralization assays using MDCKs are commonly read at 48 hours. I wonder if that would have made a difference in the results between organoids and MDCK cells.
10) Pleas specify which virus amount was used for neutralization assays.
11) Please correct minor spelling mistakes across the manuscript

Reviewer #2

(Remarks to the Author)
In the manuscript entitled 'Cattle and human organoids reveal cross-species transmission potential of 2.3.4.4b H5N1 and neuraminidase-specific neutralizing antibodies in human, the authors, Li et al., establish bovine organoids from lung and mammary gland tissue in parallel with human lung and nasal mucosa organoids to study, in particular, the differences in infection between influenza virus strains, including 2.3.4.4b H5N1, which is spreading among cattle. Cross-contamination between cattle and humans therefore represents a potential risk of zoonosis, and having in vitro models such as organoids to study the interactions of this virus with target cells in a comparative manner between tissues and species represents a real step forward. Overall, the manuscript is very comprehensive and interesting, highlighting the hypothesis that NA antibodies provide significant protection against this highly pathogenic virus in humans.
However, a number of points need to be clarified, as many methodological descriptions are insufficiently detailed and lack precision. Generally, although references are made to protocols for establishing, characterising and infecting organoids, these references are indirect, by referring to one publication and then another, etc….(lines 368, line 370, line 372). Why not cite the first one that is a reference in the field, particularly on methodology!. This process lacks transparency and is not satisfactory for understanding the establishment of organoids – particularly bovine organoids – and their infection. Line 367, reference 20 does not specify the BiomOrgan number in the suppliers. Why not cite the original paper? What shortcut are the authors proposing?
Furthermore, lines 98-99: What does "Optimized medium supplemented with growth factors" mean? Is it the medium described (reference 27) or a modification of it? But which one? This description remains very vague.
How many animals were sampled and at what age? Were they lactating animals? Nothing is indicated about comparisons and the robustness of the protocol between animals —line 369. References for antibodies CC10, ACCTUB, MUC5AC, etc. are not provided, nor is there any demonstration of a human v. bovine cross-reaction. This is not automatic!
All of this methodological data is sloppy and does not inspire confidence in certain data.
Another critical point is the use of MOI as an indicator of infection. By definition, MOI is the number of viral particles divided by the number of target cells. However, throughout the manuscript, there is no reference to the number of cells in any of the systems used - hAWO, cAwO, CMO, hhNsO – no indication of dissociation and therefore no assessment of the number of cells is given, especially since there may still be a real difference between the total number of cells and the number of target cells, as shown by the data presented in Figures 1, 2 and 3. Consequently, it is necessary to change this formulation to a number of PFUs used in the infection experiments and to clarify the process.
Although the differences between IAV and IDV viruses are clearly mentioned (lines 115-117), it is not clear which virus the hAwO are infected with as a control, type 1 or type D.
It is mentioned that "luminal cells were the primary target of cattle H5N1 virus" (line 102). However, the CMOs as illustrated (Figure 1F-G) have the lumen on the inside, and there is no mention of an in-out approach to access the inner epithelial border? Therefore, in the infection of CMOs, the luminal pole is even not in in an opened configuration. It is unclear how the infectious process is reproduced (line 132). So difficult to understand why and how the 'cattle mammary organoids adequately recapitulated the host-specificity of IDV infection' lines 131-133. How is the concept of host target cell specificity understood? It is not clear at all which cell type is the target cell - Figure 2E, as the labelling is exclusively external. There is thus a lack of detail on the validity of this model for mimicking internal infection of the mammary gland.
Detail line 650 'with 2.3.3.4b H5N1 - typographical error?
It is mentioned that ciliated cells are depleted in infected wells (line 151, Fig. 2B). In this same figure, does the number of NP+ cells correspond to the intensity of this depletion—especially for H5N1-C—as expected? Can the authors comment on and modify the text? Similarly, in Figures 3G and 3H, there is a very significant discrepancy between the ACCTUB and NP labellings. This also does not seem to be consistent with the percentages given by the FACS analysis (Fig. 3E, 3F), particularly between H5N1-C and H5NA-VN1194, which goes from 22.2% to 5.67%, whereas the cellular surface appears to be completely depleted by the second virus! Please comment and modify accordingly.
The data presented in Fig. 5 are convincing, but as suggested by the authors, it will be interesting to validate the approach using a larger series of donors.
In conclusion, while the observations in the manuscript are very interesting, a lot of details should be provided to reinforce the credibility of the data presented.

Reviewer #3

(Remarks to the Author)
Li et al. describe the development of respiratory and mammary cattle organoids and use these models to study the tropism of recent H5N1 clade 2.3.4.4b viruses in cattle. They compared the replication capacity of clade 2.3.4.4b H5N1 viruses in cattle organoids with that of an older 2004 H5N1 virus and a human H1N1 virus. They also showed that clade 2.3.4.4b H5N1 viruses tend to replicate more efficiently than the older H5N1 strain in human airway organoids. Finally, they developed a novel neutralization assay using human airway organoids that accounts for neuraminidase specific antibody activity at the viral entry stage by inhibiting neuraminidase mediated mucus penetration.
Overall, this article by Li et al. is very well written and pleasent to read. The experiments are carefully executed, and the conclusions are well supported by the data. However, several points would benefit from further attention—particularly placing the findings in the context of prior work and acknowledging earlier contributions in the field. In addition, the study remains largely descriptive and does not fully leverage the organoid systems to dissect the mechanistic basis underlying the increased replication fitness of clade 2.3.4.4b H5N1 viruses compared with older H5N1 strains.

Major comments:

1. Page 4: “Fortunately, unlike the severe disease and high fatality rate of human infections caused by the ancestral H5N1 lineages7,13, human 2.3.4.4b virus infection in the U.S. is unexpectedly mild, which also notably differs from the highly virulent and systematic infections observed in mice and ferrets after intranasal inoculation with the bovine H5N1 virus14.”
Page 11: “Despite the widespread nature of 2.3.3.4b H5N1 viruses in cattle and other mammals, the number of confirmed human infections remained comparatively low, and most human infection cases present with mild symptoms7,8. As such, the high replication of 2.3.3.4b H5N1 in the human airway and nasal organoids (Fig. 3) appeared to contradict the low incidence of human infections.”

I think that authors should be more careful with directly inferring from the situation in the US that the recent clade 2.3.4.4b H5N1 viruses are intrinsically causing a milder disease than older H5N1 viruses. It is very likely that mild human cases with ancestral H5 viruses have been under-estimated and under-reported. This is supported by serological studies reporting H5 seropositivity of poultry workers in Asia and Africa. For the same reasons, one cannot affirm that the incidence of infections with clade 2.3.4.4b viruses is much lower than that of ancestral viruses. In addition, there are other factors potentially explaining the different disease presentation in recent human cases detected in the US, such as the different route of inoculation (ocular route through indirect contact with contaminated milk versus respiratory in a dusty contaminated environment such as poultry barns and live poultry markets). The authors bring forward an interesting alternative hypothesis: the presence of cross-reactive N1 antibodies due to past exposure to H1N1 viruses would blunt disease severity. However, there is evidence that these N1 antibodies also cross-react with older H5N1 viruses (see below). Testing the Vietnam H5N1 virus in the neutralization assay would help addressing this point.
2. The discussion is mostly a repetition of the results and fails to contrast the work presented in this study with the literature. Figures are cited again in the discussion, which is uncommon. One would have liked to read elements putting the work in context with what has already been published on the replication of H5N1 viruses in cattle culture systems or in vivo, and perhaps to see more discussion bringing forward hypotheses as to why clade 2.3.4.4b viruses show increased replication in cattle organoids as compared to older H5N1 viruses or human viruses.
a. Cattle mammary gland organoids have been previously described and used to study the replication and tropism of clade 2.3.4.4b viruses (e.g. Kim et al., Science Advances, 2025), and several other articles have studied the replication of clade 2.3.4.4b H5N1 viruses in other mammary glands tissue culture systems (e.g. Turnbull et al, Nature Communications, 2025). Unfortunately, the results of the present study were not put into context with the data from these previous studies.
b. While the authors only refer to one study (ref 2), it has been already reported in other studies that N1 antibodies induced by seasonal H1N1 infection or vaccination in people can neutralize recent clade 2.3.4.4b H5N1 viruses (e.g. Daulagala et al., EID, 2024; Power et al., MedRixv https://doi.org/10.1101/2025.07.31.25331995) or even older H5N1 viruses (e.g. Sandbulte, Plos Med 2007). These studies (and others) should be acknowledged and discussed.
3. Methods lack sufficient details to fully evaluate the work presented in the article. In particular, the procedures for the culture of cattle respiratory and mammary gland organoids should be described in detail. How were the mammary gland organoids infected? The susceptible cells are the luminal cells, which should be located inside the organoids. Were the organoids first mechanically disrupted before inoculation? Strikingly, the infected cells seem to be at the periphery of the organoid in Figure 2E. The neutralization assay also deserves a more detailed procedure: in how many replicates was it performed (one can expect variation per transwell in the amount of mucus produced depending on the differentiation level and number of goblet cells)? Why was it not performed at the air-liquid interface? How many serum dilutions were tested? Were the virus controls, without sera, also serially diluted to help assessing the 50% inhibition point? How were the data transformed and normalized from Ct values to % of inhibition?
4. Terminology used to refer to the different type of organoids should be more precise. For the respiratory organoids, the immature organoids are dissociated and further differentiated at the air-liquid interface leading to a differentiated multilayered epithelium. In contrast, the mammary organoids were directly differentiated with enrichment of luminal cells (using EGF) and kept as an organoid structure. Yet the authors refer to the two systems interchangeably as organoids, or sometimes use the word monolayer to describe the airway organoids, while these organoid derived epithelial cell layers are composed of several layers. It would be clearer if different terms were used to differentiate them, e.g. “organoid-derived differentiated 2D cultures” and “organoids”.
5. The authors use both human nasal and airway organoids. The term “airway” is vague, technically, nasal organoids are also airway organoids. Please specify the anatomical region from which the airway organoids were derived (bronchus, lung).

Minor comments:
1. Figure 1C shows two panels probably because MUC5AC+ goblet cells and CC10+ club cells could not be stained simultaneously. However it is unclear whether the two pictures are from the same differentiated layer.
2. Figure 1H, S1, Figure 2 and 3: on which cells were the TCID50/plaque assays performed?
3. H5N1-VN1194 replicated less robustly, which was verified in airway organoids derived from a second donor (Fig. 3B). However, differences were less pronounced than for donor 1 and less significant, please revise text to highlight these differences.
4. For all figures, please indicate whether the replicates are technical or biological. For Fig 5 (and other figures), “Data for each experiment were obtained from a representative neutralization experiment (n=4)” is unclear. Does it mean that the experiment was done 4 times but just one experiment is shown?
5. Fig 5B: the curve of the triangle sera does not look like a neutralization curve. Can the authors explain?

Version 1:

Reviewer comments:

Reviewer #2

(Remarks to the Author)
Most of the points raised were addressed, and the authors responded satisfactorily to all the comments, particularly regarding methodology – such as the infection of organoids.

Reviewer #3

(Remarks to the Author)
The authors have substantially improved the manuscript and addressed most of my comments. However, some imprecisions remain:

1. Line 295: “Ex vivo udder explants were also used to demonstrate 2.3.4.4b H5N1 mammary infection47, yet these explants are not readily accessible and have an inherent issue of tissue heterogeneity”. It is not clear what the authors means with “explants are not readily accessible”. Please precise.

2. Line 300-303: “The enhanced replication fitness of 2.3.4.4b H5N1 relative to the ancestral H5N1-VN1194 might be related to accumulated mutations, especially in PB2, which enhances viral polymerase activity in mammalian cells47,48.” This sentence was added in response to my second comment concerning the scope of the discussion and my suggestion to propose hypotheses explaining the replication advantage of clade 2.3.4.4b H5 viruses in cattle organoids relative to older H5N1 or human isolates. However, the statement would benefit from greater precision. In particular, H5N1 1194 already carries the PB2 E627K substitution, which weakens the contrast being made. It may therefore be more appropriate to hypothesize that H5N1 viruses isolated from dairy cattle harbor distinct PB2 substitutions that enhance interactions specifically with bovine ANP32 proteins—such as M631L—as suggested by Dholakia et al.

3. Update the preprints to the published peer-reviewed articles in the reference list when possible.

4. I commend the authors for providing more details in the methods, yet some imprecisions persist:

a. Line 403: “After rinsing with a basal medium (Advanced DMEM/F-12 (Gibco), 1% HEPES, 1% GlutaMAX, and 1% Penicillin/Streptomycin), airway and nasal organoids were inoculated with indicated viruses at the indicated MOI for 2 hours, followed by incubation in the basal medium at 37°C17,24.” The MOI are not indicated. What does rinsing means? Were both the apical and basolateral media removed? With what buffer were the washes performed?

b. Line 440: “H5N1-C (0.01 MOI), (H1N1)pdm09 (0.01MOI) and H5N1-VN1194 (0.01MOI) were mixed with serially diluted human sera in 96-well plates. Each serum sample was prepared using 10 serial 3-fold dilutions, ranging from an initial dilution of 1:10 down to 1:196,830. After incubation at 37°C for 1 hour, the virus-serum mixture was then transferred to human airway organoids in 96-well transwell inserts, or MDCK cells seeded in 96-well plates, and incubated at 37°C for an additional 2 hours.” This sentence suggests that the apical medium was not removed to preserve mucus in the apical compartment, but this point is not clearly explained. Moreover, if the apical medium was retained, the authors should clarify the volume of virus or sera added to the apical compartment.
c. How was the number of cells available for infection determined to calculate the MOI? In an answer to another reviewer, the authors wrote: We always calculate the cell number after enzymatically dissociating the organoids into single cells, which is used to determine the MOI for inoculation. However, this information has not been added in the methods.
d. Thank you for providing the inhibition rate calculatation in the rebuttal. Please include it in the methods

5. Minor comment number 4 was not sufficiently answered. In most figure legends, the authors indicate: “Data represent mean and SD of the indicated number (n) of biological replicates from a representative experiment. The experiment was independently performed three times with similar results.” If replicates are performed in one experiment, then they are technical replicates and not biological replicates. The data should show the average and variation of the three biological replicates. A statement saying that the results were similar between the three biological independent replicates is not sufficient.

6. Line 304: “Despite the widespread nature of 2.3.4.4b H5N1 viruses in cattle and other mammals, clinically confirmed human infections remain comparatively limited, mostly presenting with mild symptoms7,8, although the low incidence might be attributed to under-reporting49. Nonetheless, the high replication of 2.3.4.4b H5N1 in human airway and nasal organoids appeared to contradict the mild disease in human infections.” I disagree with the assertion that high levels of replication in human airway organoids contradict the mild disease observed in humans. Viral replication in isolated cell culture systems cannot fully recapitulate disease outcomes in humans, not only because of the absence of an immune component, but also because such systems do not reproduce the infectious dose, the anatomical site of primary infection, or subsequent dissemination throughout the respiratory tract.

Open Access This Peer Review File is licensed under a Creative Commons Attribution 4.0 International License, which permits use, sharing, adaptation, distribution and reproduction in any medium or format, as long as you give appropriate credit to the original author(s) and the source, provide a link to the Creative Commons license, and indicate if changes were made.
In cases where reviewers are anonymous, credit should be given to 'Anonymous Referee' and the source.
The images or other third party material in this Peer Review File are included in the article's Creative Commons license, unless indicated otherwise in a credit line to the material. If material is not included in the article's Creative Commons license and your intended use is not permitted by statutory regulation or exceeds the permitted use, you will need to obtain permission directly from the copyright holder.
To view a copy of this license, visit https://creativecommons.org/licenses/by/4.0/

Dear reviewers,

We appreciate your encouraging and constructive comments and suggestions, which let us to improve the paper substantially.

We have addressed each of your comments and inquiries point-by-point and revised the manuscript accordingly. In response to the suggestion of reviewer 2, we conducted an organoid-based neutralization assay against the ancestral H5N1-VN1194 strain, and the results are now included in the revised version of the manuscript.

Thank you for sharing your valuable insights and for your guidance in enhancing our work. We look forward to your favorable response.

Reviewer #1

The article titled "Cattle and human organoids reveal cross-species transmission potential of 2.3.4.4b H5N1 and neuraminidase-specific neutralizing antibodies in humans" is an experimental study aimed at developing and testing cattle airway and mammary organoids against HPAI 2.3.4.4b H5N1 virus in comparison with human airway and nasal organoid susceptibility. Additionally, the authors examined the impact of pre-existing cross-reactive NA antibodies in human organoids neutralization assays with 2.3.4.4b virus.

The article is well written and contains novel and impactful information regarding influenza virus replication and cell tropism.

The reviewer has a few minor revisions that would benefit the manuscript:

1) Starting from the title and throughout the manuscript, the authors mention concepts such as "transmission", "protection from infection", etc. these are claims that the authors cannot prove with just the experiments listed within the manuscript. I would revise the authors' title and conclusions.

Thank you for your encouraging and insightful comments. We agree that our study does not directly demonstrate in vivo transmission or protection from infection. However, we examined 2.3.4.4b H5N1 infection and replication kinetics in biologically active cattle and human organoids, which could help us to extrapolate human and cattle susceptibility to the virus (1). The neuraminidase-specific antibody mediated "protection from infection" is supported by the novel organoid-based neutralization assay (Fig. 5), which directly measures antibody-mediated inhibition of viral replication (a key correlate of host protection against influenza infection) in human airway

organoids, a physiologically relevant model of human airway epithelium. In real-world infections, host susceptibility is mostly determined by viral replicative fitness and host protection from the virus. In this study, we examined these two components in biologically relevant organoid models, which, we believe, can extrapolate virus transmission potential and protection from infection.

2) The methods cited for the generation of the cattle airway and mammary gland organoids are not specific for cattle. Given the radically different anatomy between human and cattle, and the fact that both the mammary gland and airways cell types, receptor distribution and structures vary considerably depending on the collection site, production/not in production stage, age of the animal, etc. I would strongly recommend adding those specific details to the materials and methods of manuscript.

We appreciate the reviewer's comment. Indeed, we generated cattle airway and mammary gland organoids, building upon the techniques of deriving human respiratory and breast organoids, respectively (2-5). These cattle and human organoids belong to an overarching category of adult stem cell (ASC)-derived epithelial organoids. Deriving these epithelial organoids is to leverage the intrinsic attributes of ASCs for tissue repair, which is shared very likely across mammals and non-mammals. Apart from human organoids, we and others have established robust protocols to efficiently derive epithelial organoids from various species, including bats and snakes (6-8).

1) A plethora of research has established that these ASC-derived organoids retain the genetic background, gene expression profile, cellular composition and some functionality of the original tissues (9, 10). More importantly, these epithelial organoids maintain high stability over prolonged expansion culture. We characterized the cellular composition in cattle airway and mammary organoids (Fig. 1C, G). Moreover, the cattle airway organoids recapitulate the susceptibility of cattle to influenza D viruses (Fig. 1H), further verifying organoids' high biological relevance to the native cattle airway epithelium. As such, it is reasonable to extrapolate that these cattle organoids retain the receptor distribution as the cattle tissues from which the organoids are derived.

2) We did not collect the tissues from specific sites in the cattle lung and udder. Actually, we don't have to do so. The cocktail medium selectively sustains the growth of tissue-resident ASCs in the cattle airway and mammary alveolar epithelium, similar to that for deriving human airway and breast organoids (2-4). The derived organoids morphologically and functionally mimic cattle airway and mammary alveolar epithelium, both of which are susceptible to cattle H5N1 2.3.4.4b viruses (11, 12).

3) As per your suggestion, we have specified in the methods section (page 16, line 387) that the tissues were collected from a 2-year-old lactating Holstein-Friesian cow.

3) After EGF modifications, the CMO microscopically appear rather to be inverted (epithelial lining on the outer side) while also maintaining some minimal intra-luminal invaginations lined by squamous epithelium. This appears evident in both Figure 1F and 2E. Please add these details to the manuscript.

We appreciate your time and effort in reviewing the figures meticulously. In Fig. 1F, the organoids were in an apical-in polarity since these mammary organoids were cultured in 3D within Matrigel. Epithelial organoids embedded in Matrigel invariably show an apical-in polarity (13, 14). As such, the secretion produced by luminal cells is trapped within the organoids, unlike in vivo mammary alveoli, where the secretion from luminal cells is drained out. Indeed, pinkish proteinaceous secretion is discernible within the lumen of some organoids (Fig. 1E). Due to the accumulation of secretion, the luminal cells within the organoids were compressed and became deteriorated, showing a morphology similar to squamous cells, which differs from the mammary alveoli in authentic mammary tissues. Nonetheless, this seemingly apical-out polarity, and luminal cells with a "squamous cell" morphology are artificial effects related to organoid culture. We have elaborated on this on page 6 and highlighted the secretion within the CMOs in Fig. 1F.

In Fig. 2E, the organoids were indeed inverted with an apical-out polarity. The cattle H5N1 viruses infected the luminal cells in lactating cows (15), whilst the organoids' apical-in polarity would restrict the luminal cells within the intact CMOs from being accessible to the virus inoculum. To solve this problem, we mechanically disrupted the intact CMOs, followed by virus inoculation. The inoculated organoids were then cultured in suspension, rather than wrapped in Matrigel. As aforementioned, the epithelial organoids would become an apical-out polarity if not embedded in Matrigel (13, 14). Another consideration to choose suspension culture to enable an apical-out polarity after virus inoculation is that this enabled the infected luminal cells to freely release progeny virions, thus facilitating viral replication. Overall, the organoids in Fig. 2E are indeed apical-out. The infected luminal cells are located in the exterior of organoids. We have revised the manuscript to have a detailed description of these results and the infection methods on pages 8 and 17.

4) At histology, the cattle mammary alveoli are involuted (not secreting) while at EM of the CMOs the authors sustain that they see milk production. Please explain this discrepancy.

As aforementioned, we believe that CMOs in Fig. 1F show some artificial effects due to Matrigel-based organoid culture. Nonetheless, these CMOs accommodate secretory luminal cells and basal cells, similar to human breast organoids that have been well-

characterized (2, 3). Moreover, through TEM, we have identified characteristic organelles (Fig. 1G) specific to native mammary luminal cells. The presence of these ultra-structures evidences that the derived CMOs contain biologically relevant luminal cells. Secretions from luminal cells are also clearly discernible in some CMOs (Fig. 1F).

5) Given that the organoids were developed from inactive mammary glands, while most the outbreak were from lactating cattles, could the authors comment on the possibility to develop fully active/secreting mammary alveoli and whether milk production would impact virus replication.

As we have specified above, the CMOs were derived from the mammary tissues of a lactating cow. Although the histological images have some artificial effects, the derived CMOs do contain luminal cells, which are the cell population susceptible to H5N1 2.3.4.4b (15). Presumably, milk production and virus replication may affect each other, which definitely warrants our further investigation in the future.

6) Line 95 – Cuboidal cells lining the alveoli of the mammary gland are intrinsically different from the cells lining the ducts and secretory portions. Alveolar cells are secretory while ductular cells are not. Please revisit.

Thank you for your comments. We have revised the description on page 6, line 99 accordingly.

7) It would be interesting to confirm whether the high replication in organoids can be explained by the proportion and cell types present in culture, and whether that differs significantly from the original in vivo sample. Given that IF markers for cattle are limited, my recommendation is to perform single cell sequencing analysis. This will allow proper characterization of the cell types present, their transcriptomic profiles and their frequencies.

Thank you for this insightful suggestion. We have demonstrated that the cattle organoids contain the major cell populations in native tissues (Fig. 1C, G) and cattle airway organoids adequately reproduce real-world infections of cattle-specific influenza D virus (Fig. 1H). With these results, we feel confident to claim that high viral replication in these epithelial organoids stems from the high biological relevance (containing diverse cell types with comparable proportions to those in native epithelia in vivo), which has been repeatedly testified in human epithelial organoids (4, 16-18). The productive infection of H5N1 2.3.4.4b in cattle organoids indeed recapitulated, rather than differed from, the real-world infection in cattle mammary glands and respiratory tract (11, 12, 15).

We agree that conducting a scRNA-seq analysis will enable a more detailed

characterization of cattle organoids and the transcriptional profiles. We appreciate your suggestion and will incorporate this into our future research.

8) The reviewer suggests the use of Alcian blue histochemistry and/or IF for the specific visualization of mucins. PAS is less specific and overlaps with cytoplasmic polysaccharides and glycoproteins/lipids. Mucin IF, instead is more specific for mucin glycoproteins and various markers are available (e.g., MUC5AC, MUC5B, MUC2).

We appreciate the reviewer's comment. We agree that Alcian blue histochemistry will enhance the specificity of mucin characterization in our organoid. Actually, we have conducted bulk and single-cell RNA sequencing analysis in these human airway organoids (18). The results showed that the airway organoids highly expressed various goblet cell markers, including MUC5AC, MUC15, and MUC2. Moreover, we have repeatedly demonstrated the presence of MUC5AC+ goblet cells in the airway organoids (4, 18). We now include an image of MUC5AC+ goblet cells in human airway organoids as Supplementary Fig. 3. Overall, the current results demonstrate that goblet cells secrete mucins and form a mucus layer on top of airway organoid monolayers.

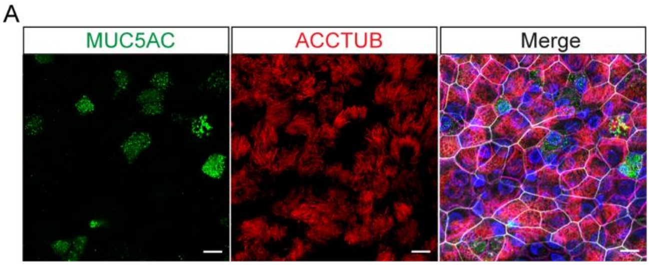

Supplementary Fig. 3. Confocal imaging of MUC5AC+ goblet cells in human airway organoids. Confocal images of MUC5AC+ goblet cells (green) and ACCTUB+ ciliated cells (red) in human airway organoids. Nuclei and actin filaments were counterstained with DAPI (blue) and Phalloidin-647 (white), respectively. Scale bar, 10 µm.

9) Please explain why in the VN, viral loads were assessed by PCR instead of HA or virus titration? Also, most microneutralization assays using MDCKs are commonly read at 48 hours. I wonder if that would have made a difference in the results between organoids and MDCK cells.

Most MN assays using MDCK cells are indeed assessed at 48 hours post-infection, as this is typically sufficient to observe cytopathic effects (CPE) and calculate the neutralization titre. However, airway organoids do not show obvious CPE after virus infections, since basal cells proliferate to replace infected and damaged cells. Therefore,

we chose to utilize qPCR-based quantification of viral load to measure viral replication and antibody-mediated neutralization in organoids and MDCK cells, which allowed a direct comparison of the two models. Since the same RT-qPCR-based viral load detection was used, the difference in NT50 readouts between organoids and MDCK cells would stem from organoid/cell themselves. Actually, it was our hypothesis that NT50 readouts would be different in organoids and MDCK cells; the airway organoid-based neutralization assay could **exclusively** reveal the real-world scenario of NA antibody-mediated protection from virus infection since airway organoids contain mucin-secreting goblet cells, forming a unique and biologically relevant mucus layer, where NA-directed antibodies take action.

10) Pleas specify which virus amount was used for neutralization assays.

MOI 0.01 is used for neutralization assays for both H1N1pdm and H5N1-C in MDCK and organoid. We have specified this in the methods section of the manuscript on page 18, line 447.

11) Please correct minor spelling mistakes across the manuscript

We have checked carefully and corrected typos and errors in the manuscript.

Reviewer #2:

In the manuscript entitled 'Cattle and human organoids reveal cross-species transmission potential of 2.3.4.4b H5N1 and neuraminidase-specific neutralizing antibodies in human, the authors, Li et al., establish bovine organoids from lung and mammary gland tissue in parallel with human lung and nasal mucosa organoids to study, in particular, the differences in infection between influenza virus strains, including 2.3.4.4b H5N1, which is spreading among cattle. Cross-contamination between cattle and humans therefore represents a potential risk of zoonosis, and having in vitro models such as organoids to study the interactions of this virus with target cells in a comparative manner between tissues and species represents a real step forward. Overall, the manuscript is very comprehensive and interesting, highlighting the hypothesis that NA antibodies provide significant protection against this highly pathogenic virus in humans.

However, a number of points need to be clarified, as many methodological descriptions are insufficiently detailed and lack precision. Generally, although references are made to protocols for establishing, characterising and infecting organoids, these references are indirect, by referring to one publication and then another, etc….(lines 368, line 370, line 372). Why not cite the first one that is a reference in the field, particularly on

methodology!. This process lacks transparency and is not satisfactory for understanding the establishment of organoids – particularly bovine organoids – and their infection. Line 367, reference 20 does not specify the BiomOrgan number in the suppliers. Why not cite the original paper? What shortcut are the authors proposing?

We appreciate the reviewer's comment. The references have been updated. Additionally, we have prepared detailed protocols for establishing cattle organoids in the Methods section on page 16. The catalogue number for the respiratory organoid expansion medium from BiomOrgan is also provided.

We are not proposing any shortcut. After working with organoids for over 10 years, organoid culture has become a routine in our lab. So we did not describe the protocols in detail. We apologize for this.

Furthermore, lines 98-99: What does "Optimized medium supplemented with growth factors" mean? Is it the medium described (reference 27) or a modification of it? But which one? This description remains very vague.

We have revised this section and prepared a Supplementary Table 1, in which we list all the components in the medium.

How many animals were sampled and at what age? Were they lactating animals? Nothing is indicated about comparisons and the robustness of the protocol between animals —line 369. References for antibodies CC10, ACCTUB, MUC5AC, etc. are not provided, nor is there any demonstration of a human v. bovine cross-reaction. This is not automatic!

We collected lung and mammary tissues from a two-year-old lactating Holstein-Friesian cow. We generated cattle airway and mammary gland organoids based on the protocols for deriving human airway and breast organoids, respectively (2-5). These cattle and human organoids belong to an overarching category of adult stem cell (ASC)-derived epithelial organoids. The rationale for deriving these epithelial organoids is to leverage the intrinsic attributes of ASCs for tissue repair, which are very likely shared across humans and animals of different species. Thus, building on techniques for deriving human organoids, we and others have established robust protocols to efficiently derive epithelial organoids from various species, including bats and snakes (6-8). We have provided detailed protocols for cattle mammary and airway organoids establishment in the method section.

The product information of the antibodies CC10 (Proteintech, 10490-1-AP), ACCTUB (Sigma, T7941), and MUC5AC (RayBiotech, 188-10578-2) is provided in the methodology section of the revised manuscript (Supplementary Table 2). These

antibodies show cross-reactivity between humans and cattle as specified by the provider.

All of this methodological data is sloppy and does not inspire confidence in certain data.

We apologize for the inadequate description of methodologies. We have revised the methodology section to include more detailed information as per your advice.

Another critical point is the use of MOI as an indicator of infection. By definition, MOI is the number of viral particles divided by the number of target cells. However, throughout the manuscript, there is no reference to the number of cells in any of the systems used - hAWO, cAwO, CMO, hhNsO – no indication of dissociation and therefore no assessment of the number of cells is given, especially since there may still be a real difference between the total number of cells and the number of target cells, as shown by the data presented in Figures 1, 2 and 3. Consequently, it is necessary to change this formulation to a number of PFUs used in the infection experiments and to clarify the process.

We appreciate the reviewer's comment. We have described the differentiation protocols for generating mature human airway and nasal organoid (hAwO and hNsO) monolayers previously (4, 19). The cAwO monolayers were differentiated using the same protocol. The differentiated hAwO, hNsO, and cAwO monolayers were cultured in 24-well transwell inserts with 2.5 x 10^5 cells per well. We used MOI to indicate the number of virions (in PFU) per cell for inoculation based on the cell number in each transwell insert. We have clarified this in the methods section.

We have been working on respiratory organoids for many years; these organoid models, which have been fully characterized, maintain high consistency in our hands. Ciliated cells, the major cell population in the airway epithelium and also the primary target of most respiratory viruses, are notably enriched in the hAwO (4) and hNsO (19), and current cAwO (Fig. 1C), and maintain a consistent percentage from batch to batch before we use these organoids for various experiments.

The cattle mammary organoids (CMOs) were propagated and differentiated in 3D format within Matrigel. Before virus inoculation, the differentiated CMOs were released from the Matrigel, then mechanically sheared to expose the luminal surface and subsequently incubated with the indicated viruses with an MOI of 0.1. We always calculate the cell number after enzymatically dissociating the organoids into single cells, which is used to determine the MOI for inoculation. The detailed information for viral inoculation has been supplemented in the methodology section on page 17.

Although the differences between IAV and IDV viruses are clearly mentioned (lines 115-117), it is not clear which virus the hAwO are infected with as a control, type 1 or

type D.

The human airway organoids (hAwO) were inoculated with IDV as a negative control since humans are not susceptible to IDV. The manuscript has been revised to clarify this description on page 7, line 125.

It is mentioned that “luminal cells were the primary target of cattle H5N1 virus” (line 102). However, the CMOs as illustrated (Figure 1F-G) have the lumen on the inside, and there is no mention of an in-out approach to access the inner epithelial border? Therefore, in the infection of CMOs, the luminal pole is even not in in an opened configuration. It is unclear how the infectious process is reproduced (line 132). So difficult to understand why and how the 'cattle mammary organoids adequately recapitulated the host-specificity of IDV infection’ lines 131-133. How is the concept of host target cell specificity understood? It is not clear at all which cell type is the target cell - Figure 2E, as the labelling is exclusively external. There is thus a lack of detail on the validity of this model for mimicking internal infection of the mammary gland.

We apologize for the inadequate description of the methodology. We also appreciate your insightful comment on Fig. 1I, which allows us to realize that influenza D viruses primarily cause cattle respiratory infections. IDV replication in cattle airway organoids recapitulated the virus's specific host tropism and also verified the biological relevance of cattle airway organoids. Yet little evidence is currently available to show IDV mammary infection in cattle. Thus, we decided to delete Fig. 1I, which is redundant and problematic.

Next, we would like to clarify the polarity of cattle mammary organoids. Cattle mammary organoids shown in Fig. 1F were in an apical-in polarity since these mammary organoids were cultured in 3D within Matrigel. Epithelial organoids 3D cultured in Matrigel always show an apical-in polarity (13, 14) and vice versa. However, the cattle mammary organoids in Fig. 2E were indeed inverted with an apical-out polarity. The cattle H5N1 viruses infected the luminal cells in lactating cows (15), whilst the cattle mammary organoids’ apical-in polarity restricts the luminal cells from being accessible to the virus inoculum, as you have expected. To solve this problem, we mechanically disrupted the intact CMOs, followed by virus inoculation. The inoculated organoids were then cultured in suspension, rather than wrapped in Matrigel. The epithelial organoids would develop an apical-out polarity if not embedded in Matrigel as mentioned above (13, 14). Collectively, the organoids shown in Fig. 2E are indeed apical-out, with infected luminal cells located on the exterior of the organoids. We have revised the manuscript to give a detailed description of these results and the

infection methods on pages 8 and 17.

Detail line 650 'with 2.3.3.4b H5N1 - typographical error?

We corrected the typo accordingly.

It is mentioned that ciliated cells are depleted in infected wells (line 151, Fig. 2B). In this same figure, does the number of NP+ cells correspond to the intensity of this depletion—especially for H5N1-C—as expected? Can the authors comment on and modify the text? Similarly, in Figures 3G and 3H, there is a very significant discrepancy between the ACCTUB and NP labellings. This also does not seem to be consistent with the percentages given by the FACS analysis (Fig. 3E, 3F), particularly between H5N1-C and H5NA-VN1194, which goes from 22.2% to 5.67%, whereas the cellular surface appears to be completely depleted by the second virus! Please comment and modify accordingly.

We appreciate the reviewer's insightful comments. We observed that ACCTUB+ ciliated cells were markedly depleted in virus-infected organoids. Moreover, ciliary depletion was more pronounced in organoids infected with H5N1-C compared to those infected with H5N1-VN1194. The more intensive the infection, the fewer cilia in the organoids. This is a common observation in airway organoid infections of other respiratory viruses reported by us and others (16, 18-20). We previously demonstrated a reduced percentage of ACCTUB+ ciliated cells in SARS-CoV-2-infected airway organoids quantitatively by flow cytometry analysis (19).

In Fig. 3, the virus-infected cells, i.e., NP+ cells, were quantified by flow cytometry analysis, while confocal imaging was performed after co-staining of NP+ cells and ACCTUB+ cilia. Cilia are hair-like projections on ciliated cells that take a thin column shape. NP signals are better revealed in the cytoplasm, where cilia are not fully manifested. As such, single-layer images (shown in Fig. 3G and 3H) are quite difficult to show both markers properly. Anyway, we appreciate the reviewer's comment that fewer cilia in H5N1-1194-infected organoids than in H5N1-C-infected organoids (in Fig. 3G and 3H) does not align with our description and interpretation. We now replace them with more representative confocal images that were captured from different areas in the same slides.

The data presented in Fig. 5 are convincing, but as suggested by the authors, it will be interesting to validate the approach using a larger series of donors.

We agree with the reviewer that our approach warrants a validation with a larger series of organoids from more donors in the future.

In conclusion, while the observations in the manuscript are very interesting, a lot of details should be provided to reinforce the credibility of the data presented.

We appreciate the reviewer's encouraging comment. We have revised the manuscript and incorporated more details to reinforce the vigour of our study.

Reviewer #3:

Li et al. describe the development of respiratory and mammary cattle organoids and use these models to study the tropism of recent H5N1 clade 2.3.4.4b viruses in cattle. They compared the replication capacity of clade 2.3.4.4b H5N1 viruses in cattle organoids with that of an older 2004 H5N1 virus and a human H1N1 virus. They also showed that clade 2.3.4.4b H5N1 viruses tend to replicate more efficiently than the older H5N1 strain in human airway organoids. Finally, they developed a novel neutralization assay using human airway organoids that accounts for neuraminidase specific antibody activity at the viral entry stage by inhibiting neuraminidase mediated mucus penetration.

Overall, this article by Li et al. is very well written and pleasent to read. The experiments are carefully executed, and the conclusions are well supported by the data. However, several points would benefit from further attention—particularly placing the findings in the context of prior work and acknowledging earlier contributions in the field. In addition, the study remains largely descriptive and does not fully leverage the organoid systems to dissect the mechanistic basis underlying the increased replication fitness of clade 2.3.4.4b H5N1 viruses compared with older H5N1 strains.

We appreciate the reviewer's encouraging comment. We also agree with the reviewer that the mechanism underlying the higher fitness of 2.3.4.4b H5N1 viruses than older H5N1 strains has not been explored in this study. This would be an important question and definitely warrants our further investigations in the future.

Major comments:

1. Page 4: "Fortunately, unlike the severe disease and high fatality rate of human infections caused by the ancestral H5N1 lineages7,13, human 2.3.4.4b virus infection in the U.S. is unexpectedly mild, which also notably differs from the highly virulent and systematic infections observed in mice and ferrets after intranasal inoculation with the bovine H5N1 virus14."

Page 11: "Despite the widespread nature of 2.3.3.4b H5N1 viruses in cattle and other mammals, the number of confirmed human infections remained comparatively low, and most human infection cases present with mild symptoms7,8. As such, the high

replication of 2.3.3.4b H5N1 in the human airway and nasal organoids (Fig. 3) appeared to contradict the low incidence of human infections."

I think that authors should be more careful with directly inferring from the situation in the US that the recent clade 2.3.4.4b H5N1 viruses are intrinsically causing a milder disease than older H5N1 viruses. It is very likely that mild human cases with ancestral H5 viruses have been under-estimated and under-reported. This is supported by serological studies reporting H5 seropositivity of poultry workers in Asia and Africa. For the same reasons, one cannot affirm that the incidence of infections with clade 2.3.4.4b viruses is much lower than that of ancestral viruses. In addition, there are other factors potentially explaining the different disease presentation in recent human cases detected in the US, such as the different route of inoculation (ocular route through indirect contact with contaminated milk versus respiratory in a dusty contaminated environment such as poultry barns and live poultry markets). The authors bring forward an interesting alternative hypothesis: the presence of cross-reactive N1 antibodies due to past exposure to H1N1 viruses would blunt disease severity. However, there is evidence that these N1 antibodies also cross-react with older H5N1 viruses (see below). Testing the Vietnam H5N1 virus in the neutralization assay would help addressing this point.

We appreciate the reviewer's insightful comments. We also noticed the related reports and agree with the reviewer that infections of both 2.3.4.4b H5N1 and ancestral Vietnam H5N1 might be under-estimated and under-reported. We have revised the manuscript accordingly on page 4 and 13.

As per your suggestion, we measured serum antibodies and found that these N1 antibodies indeed cross-neutralized the ancestral H5N1 virus in airway organoids. The new data are presented in Fig. 5E, indicating N1 antibodies may also protect against the infection of ancestral H5N1 viruses.

2. The discussion is mostly a repetition of the results and fails to contrast the work presented in this study with the literature. Figures are cited again in the discussion, which is uncommon. One would have liked to read elements putting the work in context with what has already been published on the replication of H5N1 viruses in cattle culture systems or in vivo, and perhaps to see more discussion bringing forward hypotheses as to why clade 2.3.4.4b viruses show increased replication in cattle organoids as compared to older H5N1 viruses or human viruses.

We have revised the discussion as per your suggestion and highlighted the significance of our findings in the context of current knowledge. The higher replication fitness of 2.3.4.4b H5N1 than that of ancestral H5N1 viruses might be related to accumulated

mutations, especially in PB2, which enhance viral polymerase activity in mammalian cells (21, 22). These have been discussed in the revised manuscript on page 13.

a. Cattle mammary gland organoids have been previously described and used to study the replication and tropism of clade 2.3.4.4b viruses (e.g. Kim et al., Science Advances, 2025), and several other articles have studied the replication of clade 2.3.4.4b H5N1 viruses in other mammary glands tissue culture systems (e.g. Turnbull et al, Nature Communications, 2025). Unfortunately, the results of the present study were not put into context with the data from these previous studies.

These two papers were published after we started paper submission. Now we cite these two papers in the revised manuscript as per your suggestion. We have connections with Prof YK Choi (the last author) whose team has expertise in organoid culture. In the Science Advances paper, they only showed 2.3.4.4b H5N1 infection of bovine mammary organoid in a supplementary figure without any characterization of the derived mammary organoids. Here in this paper, we present more data, including heterogeneous cell populations in the derived cattle mammary organoids, cattle mammary organoids sustaining higher replication of 2.3.4.4b H5N1 than ancestral H5N1-VN1194, and their resistance to the human H1N1 virus.

As for the NC paper, we agree with the results of bovine tissues. We now cite this in the current manuscript. Yet we may not accept the results of bovine fibroblasts, BSF (bovine skin fibroblasts) and BNF (bovine nose fibroblasts). Fibroblasts in the interstitial tissues are normally not exposed to influenza viruses, and thus not the natural target cells; airway epithelial cells and, herein, mammary epithelial cells are. The data on influenza virus infection in fibroblasts may not have biological relevance.

We discuss these prior findings and highlight the novelty of our organoid models in the revised manuscript on page 12.

b. While the authors only refer to one study (ref 2), it has been already reported in other studies that N1 antibodies induced by seasonal H1N1 infection or vaccination in people can neutralize recent clade 2.3.4.4b H5N1 viruses (e.g. Daulagala et al., EID, 2024; Power et al., MedRixv https://doi.org/10.1101/2025.07.31.25331995) or even older H5N1 viruses (e.g. Sandbulte, Plos Med 2007). These studies (and others) should be acknowledged and discussed.

We have noticed these prior studies and now cite them in the revised manuscript on page 13, line 319. NA inhibitory antibodies against 2.3.4.4b H5N1 have been reported extensively (23-27). The strength of our study is that we developed organoid-based neutralization assays to measure the neutralizing activity of NA antibodies **for the first**

time. The conventional cell-line-based influenza neutralization assays only reveal the neutralization from HA antibodies, while our organoid-based assays demonstrate the overall neutralization from HA antibodies and NA antibodies. More importantly, organoid-based assays directly address whether/how potent the antibodies protect human respiratory cells, not any unrelated or seemingly related cell lines, from influenza viruses.

3. Methods lack sufficient details to fully evaluate the work presented in the article. In particular, the procedures for the culture of cattle respiratory and mammary gland organoids should be described in detail. How were the mammary gland organoids infected? The susceptible cells are the luminal cells, which should be located inside the organoids. Were the organoids first mechanically disrupted before inoculation? Strikingly, the infected cells seem to be at the periphery of the organoid in Figure 2E.

The neutralization assay also deserves a more detailed procedure: in how many replicates was it performed (one can expect variation per transwell in the amount of mucus produced depending on the differentiation level and number of goblet cells)? Why was it not performed at the air-liquid interface? How many serum dilutions were tested? Were the virus controls, without sera, also serially diluted to help assessing the 50% inhibition point? How were the data transformed and normalized from Ct values to % of inhibition?

We apologize for the inadequate description of methodologies. After working with organoids for many years, we have routine procedures to culture organoids and conduct virus infections in organoids. As per your suggestion, we have provided the detailed protocols in the revised manuscript.

We'd like to clarify the polarity of cattle mammary organoids first. Cattle mammary organoids shown in Fig. 1F were in an apical-in polarity since these mammary organoids were cultured in 3D within Matrigel. Epithelial organoids 3D cultured in Matrigel always show an apical-in polarity (13, 14) and vice versa. However, the cattle mammary organoids in Fig. 2E were indeed inverted with an apical-out polarity. The cattle H5N1 viruses infected the luminal cells in lactating cows (15), whilst the cattle mammary organoids' apical-in polarity restricts the luminal cells from being accessible to the virus inoculum, as you have expected. To solve this problem, we mechanically disrupted the intact CMOs, followed by virus inoculation. The inoculated organoids were then cultured in suspension, rather than wrapped in Matrigel. The epithelial organoids would develop an apical-out polarity if not embedded in Matrigel as mentioned above (13, 14). Thus, the virus-infected luminal cells are located in the periphery of the organoids. The detailed information for viral inoculation has been

supplemented in the methodology section on page 17.

As aforementioned, organoids, especially airway organoids, have been a major experimental model in our lab for many years. The whole organoid system, including the maturation state and percentages of various cell types within the organoids, has been fully characterized and maintained with high consistency and stability (4, 18). To generate mature airway organoids, we use transwell inserts with medium supplemented with DAPT, instead of no medium in the upper chamber (so-called air-liquid interface culture), since airway organoids with medium in both upper and bottom chambers yield more consistent organoids with improved differentiation status based on our observations. Consequently, the percentages of various cell types, including goblet cells, are highly consistent across different wells and batches. This reproducibility ensures that the mucus layer formed by the secretion of goblet cells remains consistent, thereby minimizing well-to-well and batch-to-batch variation. We always use differentiated airway organoids on 96-well transwell inserts with medium in both upper and bottom chambers for organoid-based neutralization assays (28).

The organoid-based neutralization assay of each serum was performed independently 2 to 3 times and yielded similar results. We tested 10 serial 3-fold dilutions of each serum, ranging from an initial dilution of 1:10 to 1:196,830. We did not serially dilute the virus control to assess a 50% inhibition, instead we established "virus-only" control wells (without sera, 4 wells) to represent the maximum viral load (i.e., 0% inhibition). RT-qPCR Ct values were converted to an inhibition rate using the 2^-ΔCt method. The inhibition rate was calculated as follows, $(1-2^{(-(Ct_{sample} - Ct_{virus\ only\ control}))}) \times 100\%$, where $Ct_{virus\ only\ control}$ represents the mean Ct value from 4 technical replicates of virus-only wells. Upon the transformation, NT50 values were determined using a 4-parameter non-linear regression analysis in GraphPad Prism 9 (GraphPad Software). The detailed procedure for the neutralization assay is also described in the methods section on page 18, line 447.

4. Terminology used to refer to the different type of organoids should be more precise. For the respiratory organoids, the immature organoids are dissociated and further differentiated at the air-liquid interface leading to a differentiated multilayered epithelium. In contrast, the mammary organoids were directly differentiated with enrichment of luminal cells (using EGF) and kept as an organoid structure. Yet the authors refer to the two systems interchangeably as organoids, or sometimes use the word monolayer to describe the airway organoids, while these organoid derived epithelial cell layers are composed of several layers. It would be clearer if different terms were used to differentiate them, e.g. “organoid-derived differentiated 2D cultures” and “organoids”.

We have established the first adult-stem-cell-based, two-phase respiratory organoid culture system (4, 18, 19). In this system, the expansion culture of the organoids, in a 3D format within Matrigel, maintains a stable and long-term expandable source; while the differentiation protocols allow us to generate physiologically active, mature respiratory organoids. The differentiated airway and nasal organoids could be 3D in Matrigel or a monolayer format grown on transwell inserts. Both formats of organoids contain four types of airway epithelial cell types, while monolayers are more mature than 3D counterparts (16, 17, 19, 28). In this study, we used monolayers of differentiated human nasal organoids, and differentiated human and cattle airway organoids grown on transwell inserts. We have specified this in the revised manuscript on page 6 and 16.

Cattle mammary organoids were cultured in 3D within Matrigel and differentiated in 3D format with EGF removal medium to enrich luminal cells (Fig. 1F-G), referred to as "3D mammary organoids".

5. The authors use both human nasal and airway organoids. The term “airway” is vague, technically, nasal organoids are also airway organoids. Please specify the anatomical region from which the airway organoids were derived (bronchus, lung).

We have established the first human respiratory organoid culture system(4, 14, 19, 29-32). Organoids are derived from adult stem cells in patients’ primary lung tissues and nasal epithelial cells from healthy donors with high efficiency and can be stably expanded for over half a year. To derive organoids, we used relatively normal lung tissues adjacent to patients’ diseased tissues. These lung tissues typically contain bronchioles of varying sizes, surrounded by alveolar sacs. After 1 to 2 passages, fibroblasts and other non-epithelial cells present in the initial culture gradually diminished. We have developed differentiation protocols to induce maturation in lung tissue-derived organoids and generate mature airway organoids that faithfully simulate the native airway epithelium(18). Apart from a noninvasive procedure to procure readily accessible nasal cells for organoid derivation, nasal organoids more adequately simulate the upper airway epithelium than airway organoids (19). The mature airway and nasal organoids contain four types of airway epithelial cells, yet nose-enriched and trachea/bronchi-enriched genes are significantly higher in differentiated nasal and airway organoids, respectively. A schematic presentation of our respiratory organoid culture system is enclosed herein for your reference.

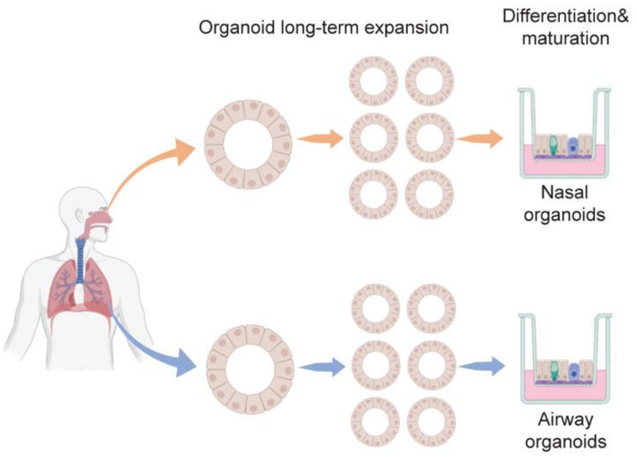

We have clarified these in the manuscript on page 5 to enhance the interpretability of our findings.

Minor comments:

1. Figure 1C shows two panels probably because MUC5AC+ goblet cells and CC10+ club cells could not be stained simultaneously. However it is unclear whether the two pictures are from the same differentiated layer.

If we co-stained MUC5AC+ goblet cells and CC10+ club together with ACCTUB+ ciliated cells, there would be five colors in the image. So we stained goblet cells and club cells separately, using the same cAwO monolayer. The membrane of transwell insert was cut into 2 halves and applied to IF staining separately. These two images look similar, but they were taken from organoid monolayers labelled with different antibodies. Moreover, we used image analysis software Proofig to check the two images and verified that they are different.

2. Figure 1H, S1, Figure 2 and 3: on which cells were the TCID50/plaque assays performed?

The viral titration was performed in MDCK cells.

3. H5N1-VN1194 replicated less robustly, which was verified in airway organoids derived from a second donor (Fig. 3B). However, differences were less pronounced than for donor 1 and less significant, please revise text to highlight these differences.

We have revised the text on page 9 to explain the variability observed between donors.

4. For all figures, please indicate whether the replicates are technical or biological. For Fig 5 (and other figures), “Data for each experiment were obtained from a representative neutralization experiment (n=4)” is unclear. Does it mean that the experiment was done 4 times but just one experiment is shown?

For Fig. 5, “4” refers to number of biological replicates.

5. Fig 5B: the curve of the triangle sera does not look like a neutralization curve. Can the authors explain?

The curve was generated using a 4-parameter non-linear regression analysis in GraphPad Prism 9. The atypical appearance of the purple curve may be attributed to the relatively poor neutralization capacity of the serum sample. We actually repeated the assays several times and got a similar result.

References:

1. Song H, Hao T, Han P, Wang H, Zhang X, Li X, et al. Receptor binding, structure, and tissue tropism of cattle-infecting H5N1 avian influenza virus hemagglutinin. Cell. 2025.
2. Sachs N, de Ligt J, Kopper O, Gogola E, Bounova G, Weeber F, et al. A Living Biobank of Breast Cancer Organoids Captures Disease Heterogeneity. Cell. 2018;172(1-2):373-86.e10.
3. Rosenbluth JM, Schackmann RCJ, Gray GK, Selfors LM, Li CM, Boedicker M, et al. Organoid cultures from normal and cancer-prone human breast tissues preserve complex epithelial lineages. Nat Commun. 2020;11(1):1711.
4. Zhou J, Li C, Sachs N, Chiu MC, Wong BH, Chu H, et al. Differentiated human airway organoids to assess infectivity of emerging influenza virus. Proc Natl Acad Sci U S A. 2018;115(26):6822-7.
5. Li C, Chiu MC, Yu Y, Liu X, Xiao D, Huang J, et al. Establishing Human Lung Organoids and Proximal Differentiation to Generate Mature Airway Organoids. J Vis Exp. 2022(181).
6. Kim H, Heo SY, Kim YI, Park D, N MPA, Hwang S, et al. Diverse bat organoids provide pathophysiological models for zoonotic viruses. Science. 2025;388(6748):756-62.
7. Post Y, Puschhof J, Beumer J, Kerkkamp HM, de Bakker MAG, Slagboom J, et al. Snake Venom Gland Organoids. Cell. 2020;180(2):233-47 e21.
8. Zhou J, Li C, Liu X, Chiu MC, Zhao X, Wang D, et al. Infection of bat and human intestinal organoids by SARS-CoV-2. Nat Med. 2020;26(7):1077-83.
9. Clevers H. Modeling Development and Disease with Organoids. Cell. 2016;165(7):1586-97.
10. Sato T, Vries RG, Snippert HJ, Van De Wetering M, Barker N, Stange DE, et al. Single Lgr5 stem cells build crypt-villus structures in vitro without a mesenchymal niche. Nature. 2009;459(7244):262-5.
11. Baker AL, Arruda B, Palmer MV, Boggiatto P, Sarlo Davila K, Buckley A, et al. Dairy cows inoculated with highly pathogenic avian influenza virus H5N1. Nature. 2025;637(8047):913-20.
12. Caserta LC, Frye EA, Butt SL, Laverack M, Nooruzzaman M, Covaleda LM, et al. Spillover of highly pathogenic avian influenza H5N1 virus to dairy cattle. Nature. 2024;634(8034):669-76.

13. Co JY, Margalef-Catala M, Li X, Mah AT, Kuo CJ, Monack DM, et al. Controlling Epithelial Polarity: A Human Enteroid Model for Host-Pathogen Interactions. Cell Rep. 2019;26(9):2509‑20 e4.
14. Chiu MC, Zhang S, Li C, Liu X, Yu Y, Huang J, et al. Apical-Out Human Airway Organoids Modeling SARS-CoV-2 Infection. Viruses. 2023;15(5).
15. Halwe NJ, Cool K, Breithaupt A, Schon J, Trujillo JD, Nooruzzaman M, et al. H5N1 clade 2.3.4.4b dynamics in experimentally infected calves and cows. Nature. 2025;637(8047):903‑12.
16. Li C, Yu Y, Wan Z, Chiu MC, Huang J, Zhang S, et al. Human respiratory organoids sustained reproducible propagation of human rhinovirus C and elucidation of virus-host interaction. Nat Commun. 2024;15(1):10772.
17. Li C, Huang J, Yu Y, Wan Z, Chiu MC, Liu X, et al. Human airway and nasal organoids reveal escalating replicative fitness of SARS-CoV-2 emerging variants. Proc Natl Acad Sci U S A. 2023;120(17):e2300376120.
18. Chiu MC, Li C, Liu X, Yu Y, Huang J, Wan Z, et al. A bipotential organoid model of respiratory epithelium recapitulates high infectivity of SARS-CoV-2 Omicron variant. Cell Discov. 2022;8(1):57.
19. Chiu MC, Li C, Liu X, Song W, Wan Z, Yu Y, et al. Human Nasal Organoids Model SARS-CoV-2 Upper Respiratory Infection and Recapitulate the Differential Infectivity of Emerging Variants. mBio. 2022;13(4):e0194422.
20. Robinot R, Hubert M, de Melo GD, Lazarini F, Bruel T, Smith N, et al. SARS-CoV-2 infection induces the dedifferentiation of multiciliated cells and impairs mucociliary clearance. Nat Commun. 2021;12(1):4354.
21. Dholakia V, Quantrill JL, Richardson SA, Pankaew N, Brown MD, Yang J, et al. Polymerase mutations underlie early adaptation of H5N1 influenza virus to dairy cattle and other mammals. Nature Communications. 2026.
22. Turnbull ML, Zakaria MK, Upfold NS, Bakshi S, Magill C, Das UR, et al. The potential of H5N1 viruses to adapt to bovine cells varies throughout evolution. Nat Commun. 2025;16(1):11042.
23. Sandbulte MR, Jimenez GS, Boon AC, Smith LR, Treanor JJ, Webby RJ. Cross-reactive neuraminidase antibodies afford partial protection against H5N1 in mice and are present in unexposed humans. PLoS Med. 2007;4(2):e59.
24. Kandeil A, Patton C, Jones JC, Jeevan T, Harrington WN, Trifkovic S, et al. Rapid evolution of A(H5N1) influenza viruses after intercontinental spread to North America. Nat Commun. 2023;14(1):3082.
25. Singh G, Bhavsar D, Hermann E, Gleason C, Singh Sandhu H, Singh P, et al. Population immunity to clade 2.3. 4.4 b H5N1 is dominated by anti-neuraminidase antibodies. medRxiv. 2026:2026.02. 10.26346014.
26. Power MA, Rijnink WF, Soochit W, Gommers L, van der Linden A, Chandler F, et al. Seasonal Influenza Exposure Elicits Functional Antibody and T-cell Responses to A (H5) Influenza Viruses in Humans. medRxiv. 2025:2025.07. 31.25331995.
27. Li ZN, Liu F, Jung YJ, Jefferson S, Holiday C, Gross FL, et al. Pre-existing cross-reactive immunity to highly pathogenic avian influenza

2.3.4.4b A(H5N1) virus in the United States. Nature Communications. 2025;16(1).
28. Wan Z, Li C, Zhou Y, Yu Y, Chiu MC, Huang J, et al. Organoid-based neutralization assays reveal a distinctive profile of SARS-CoV-2 antibodies and recapitulate the real-world efficacy. Proc Natl Acad Sci U S A. 2025;122(35):e2509616122.
29. Mallapaty S. The mini lungs and other organoids helping to beat COVID. Nature. 2021;593(7860):492–4.
30. Li C, Chiu MC, Yu Y, Liu X, Xiao D, Huang J, et al. Establishing Human Lung Organoids and Proximal Differentiation to Generate Mature Airway Organoids. J Vis Exp. 2022(181):e63684.
31. Chiu MC, Cun Li, Xiaojuan Liu, Yifei Yu, Jingjing Huang, Zhixin Wan, et al. A bipotential organoid model of respiratory epithelium recapitulates high infectivity of SARS-CoV-2 Omicron variant. Cell Discovery. 2022.
32. Chiu MC, Li C, Yu Y, Liu X, Huang J, Wan Z, et al. Establishing Bipotential Human Lung Organoid Culture System and Differentiation to Generate Mature Alveolar and Airway Organoids. Bio Protoc. 2023;13(8):e4657.

Reviewer #2:

Most of the points raised were addressed, and the authors responded satisfactorily to all the comments, particularly regarding methodology – such as the infection of organoids.

We appreciate your constructive comments and suggestions, which have allowed us to improve the paper substantially.

Reviewer #3:

The authors have substantially improved the manuscript and addressed most of my comments. However, some imprecisions remain:

1. Line 295: “Ex vivo udder explants were also used to demonstrate 2.3.4.4b H5N1 mammary infection47, yet these explants are not readily accessible and have an inherent issue of tissue heterogeneity”. It is not clear what the authors means with “explants are not readily accessible”. Please precise.

Thank you for your feedback. We intended to say that cattle tissue explants are not readily available to most research laboratories, including ours, although veterinarians or researchers in veterinary sciences may have easy access to them. We have revised the manuscript accordingly on Page 11 and highlighted it in blue.

2. Line 300-303: “The enhanced replication fitness of 2.3.4.4b H5N1 relative to the ancestral H5N1-VN1194 might be related to accumulated mutations, especially in PB2, which enhances viral polymerase activity in mammalian cells47,48.” This sentence was added in response to my second comment concerning the scope of the discussion and my suggestion to propose hypotheses explaining the replication advantage of clade 2.3.4.4b H5 viruses in cattle organoids relative to older H5N1 or human isolates. However, the statement would benefit from greater precision. In particular, H5N1 1194 already carries the PB2 E627K substitution, which weakens the contrast being made. It may therefore be more appropriate to hypothesize that H5N1 viruses isolated from dairy cattle harbor distinct PB2 substitutions that enhance interactions specifically with bovine ANP32 proteins—such as M631L—as suggested by Dholakia et al.

We appreciate the reviewer’s insightful comment. We have revised the discussion on Page 12 and highlighted that the PB2 mutation, such as M631L, enhanced replication in bovine cells and tissues, as well as in the cells of other mammals, including humans.

3. Update the preprints to the published peer-reviewed articles in the reference list when possible.

We have updated reference 38 to its published version in mBio.

4. I commend the authors for providing more details in the methods, yet some imprecisions persist:

a. Line 403: “After rinsing with a basal medium (Advanced DMEM/F-12 (Gibco), 1% HEPES, 1% GlutaMAX, and 1% Penicillin/Streptomycin), airway and nasal organoids were inoculated with indicated viruses at the indicated MOI for 2 hours, followed by incubation in the basal medium at 37°C17,24.” The MOI are not indicated. What does rinsing means? Were both the apical and basolateral media removed? With what buffer were the washes performed?

Thank you for your comment.

Regarding the MOI, since different MOIs were used for different viruses across various assays, specifying all of them in this methodology section would be cumbersome. Anyway, the specific MOI for each assay is now detailed in the corresponding Results sections and Figure legends.

Regarding the rinsing procedure, we apologize for the ambiguity. By 'rinsing', we refer to washing. Before the infection assay, media in the top and bottom chambers were removed. We performed the washings with a basal medium; its composition is specified on Page 16, line 408.

b. Line 440: “H5N1-C (0.01 MOI), (H1N1)pdm09 (0.01MOI) and H5N1-VN1194 (0.01MOI) were mixed with serially diluted human sera in 96-well plates. Each serum sample was prepared using 10 serial 3-fold dilutions, ranging from an initial dilution of 1:10 down to 1:196,830. After incubation at 37°C for 1 hour, the virus-serum mixture was then transferred to human airway organoids in 96-well transwell inserts, or MDCK cells seeded in 96-well plates, and incubated at 37°C for an additional 2 hours.” This sentence suggests that the apical medium was not removed to preserve mucus in the apical compartment, but this point is not clearly explained. Moreover, if the apical medium was retained, the authors should clarify the volume of virus or sera added to the apical compartment.

For the neutralization assay, media in the top and bottom chambers were first removed, then the organoid monolayers, both apical and basolateral sides were washed once with the basal medium. This washing step did not remove the mucus layer on the apical side. Subsequently, 150 μl of basal medium was added to the bottom chamber of each well, and 50 μl of the virus-serum mixture was added to the top chamber. We have now revised the methods section in the manuscript on Page 17.

c. How was the number of cells available for infection determined to calculate the MOI? In an answer to another reviewer, the authors wrote: We always calculate the cell

number after enzymatically dissociating the organoids into single cells, which is used to determine the MOI for inoculation. However, this information has not been added in the methods.

We have now specified this in the methods section (Page 16) as per your suggestion.

d. Thank you for providing the inhibition rate calculatation in the rebuttal. Please include it in the methods

Thank you for your constructive suggestion. We have now incorporated the calculation method for the inhibition rate into the methods section on Page 17.

5. Minor comment number 4 was not sufficiently answered. In most figure legends, the authors indicate: "Data represent mean and SD of the indicated number (n) of biological replicates from a representative experiment. The experiment was independently performed three times with similar results." If replicates are performed in one experiment, then they are technical replicates and not biological replicates. The data should show the average and variation of the three biological replicates. A statement saying that the results were similar between the three biological independent replicates is not sufficient.

We sincerely thank the reviewer for the rigorous evaluation of our statistical analysis.

First, we would like to clarify a misunderstanding regarding the description you are quoting: "The experiment was independently performed three times with similar results." Please note that this description in figure legends exclusively refers to the micrograph images; this complies with the imaging data reporting guidelines required by Nature Communications. It was not applied to describe the quantitative data graphs.

As for the issue of "biological replicate", we apologize for the inaccuracy in the last round of review when we addressed your inquiry. Here, we'd clarify that the "n=4" in Figure 5 refers to technical replicates. This is the common practice in performing these neutralization assays. However, for each serum specimen, we have performed the neutralization assays independently at least three times and obtained similar results.

6. Line 304: "Despite the widespread nature of 2.3.4.4b H5N1 viruses in cattle and other mammals, clinically confirmed human infections remain comparatively limited, mostly presenting with mild symptoms7,8, although the low incidence might be attributed to under-reporting49. Nonetheless, the high replication of 2.3.4.4b H5N1 in human airway and nasal organoids appeared to contradict the mild disease in human infections." I disagree with the assertion that high levels of replication in human airway organoids contradict the mild disease observed in humans. Viral replication in isolated

cell culture systems cannot fully recapitulate disease outcomes in humans, not only because of the absence of an immune component, but also because such systems do not reproduce the infectious dose, the anatomical site of primary infection, or subsequent dissemination throughout the respiratory tract.

We respectfully disagree with your suggestion to delete this sentence. We believe virus replication in human airway and nasal organoids can recapitulate its replication fitness in human respiratory cells, or the intrinsic susceptibility of human respiratory cells to the viruses, since these respiratory organoids faithfully simulate the native human respiratory epithelial cells. We agree with you that disease outcomes in humans are the result of the arms race between the virus and host immunity. Pre-existing neutralizing antibodies represent a major player in protecting humans from virus infections. The high replication of cattle H5N1 virus in human airway and nasal organoids versus rare and mild human infection cases prompted us to infer that pre-existing neutralizing N1 antibodies originated from prior H1N1 infections and/or flu vaccination may protect humans from cattle H5N1 infections.

As for "do not reproduce the infectious dose", we used low MOIs for examining virus replication kinetics (already specified in the legend), which may not be contradictory to real-world infections. As for anatomical site, we demonstrate infections of the indicated viruses in human nasal and airway organoids, the organoid models of upper and lower airway epithelium[1,2]. To our understanding, the upper airway epithelium is the primary infection site of many respiratory viruses. Replicating viruses may spread to the lower airways or even alveolar sacs. Indeed, we have studied these viruses in alveolar organoids[3] established in our lab in another unpublished project. Hopefully, we can share the findings with you shortly.

Reference

1 Chiu, M. C. *et al.* Human Nasal Organoids Model SARS-CoV-2 Upper Respiratory Infection and Recapitulate the Differential Infectivity of Emerging Variants. *mBio* **13**, e0194422 (2022). https://doi.org/10.1128/mbio.01944-22

2 Zhou, J. *et al.* Differentiated human airway organoids to assess infectivity of emerging influenza virus. *Proc Natl Acad Sci U S A* **115**, 6822-6827 (2018). https://doi.org/10.1073/pnas.1806308115

3 Chiu, M. C. *et al.* A bipotential organoid model of respiratory epithelium recapitulates high infectivity of SARS-CoV-2 Omicron variant. *Cell Discov* **8**, 57 (2022). https://doi.org/10.1038/s41421-022-00422-1
